# A Toolbox for Spatiotemporal Analysis of Voltage-Sensitive Dye Imaging Data in Brain Slices

**DOI:** 10.1371/journal.pone.0108686

**Published:** 2014-09-26

**Authors:** Elliot B. Bourgeois, Brian N. Johnson, Almedia J. McCoy, Lorenzo Trippa, Akiva S. Cohen, Eric D. Marsh

**Affiliations:** 1 Department of Pathology, Brigham and Women's Hospital and Harvard Medical School, Boston, Massachusetts, United States of America; 2 Department of Pediatrics, Division of Pediatric Neurology, The Children's Hospital of Philadelphia, Philadelphia, Pennsylvania, United States of America; 3 Department of Biostatistics and Computational Biology, Dana-Farber Cancer Institute, Boston, Massachusetts, United States of America; 4 Department of Neurology, Division of Pediatric Neurology, The Perelman School of Medicine at the University of Pennsylvania, Philadelphia, Pennsylvania, United States of America; Creighton University, United States of America

## Abstract

Voltage-sensitive dye imaging (VSDI) can simultaneously monitor the spatiotemporal electrical dynamics of thousands of neurons and is often used to identify functional differences in models of neurological disease. While the chief advantage of VSDI is the ability to record spatiotemporal activity, there are no tools available to visualize and statistically compare activity across the full spatiotemporal range of the VSDI dataset. Investigators commonly analyze only a subset of the data, and a majority of the dataset is routinely excluded from analysis. We have developed a software toolbox that simplifies visual inspection of VSDI data, and permits unaided statistical comparison across spatial and temporal dimensions. First, the three-dimensional VSDI dataset (x,y,time) is geometrically transformed into a two-dimensional spatiotemporal map of activity. Second, statistical comparison between groups is performed using a non-parametric permutation test. The result is a 2D map of all significant differences in both space and time. Here, we used the toolbox to identify functional differences in activity in VSDI data from acute hippocampal slices obtained from epileptic *Arx* conditional knock-out and control mice. Maps of spatiotemporal activity were produced and analyzed to identify differences in the activity evoked by stimulation of each of two axonal inputs to the hippocampus: the perforant pathway and the temporoammonic pathway. In mutant hippocampal slices, the toolbox identified a widespread decrease in spatiotemporal activity evoked by the temporoammonic pathway. No significant differences were observed in the activity evoked by the perforant pathway. The VSDI toolbox permitted us to visualize and statistically compare activity across the spatiotemporal scope of the VSDI dataset. Sampling error was minimized because the representation of the data is standardized by the toolbox. Statistical comparisons were conducted quickly, across the spatiotemporal scope of the data, without *a priori* knowledge of the character of the responses or the likely differences between them.

## Introduction

Voltage-sensitive dye imaging (VSDI) provides unparalleled resolution to assay the emergent properties of complex neural network ensembles. Transmembrane potential can be recorded with relative ease using VSDI, compared to conventional electrophysiological methods, and the spatial resolution of VSDI is limited only by the number of pixels in the VSDI camera. VSDI studies have yielded new insight into the function of native neural networks [1], [2], [3], and VSDI is increasingly applied to models of neurological disease to identify dysfunctional neural circuits [4], [5]. VSDI is typically used to record activity evoked by trains of electrical stimuli, but more recently VSDI has been used to record activation patterns that result from optogenetic stimulation [6], [7].

While the chief advantage of VSDI is the ability to broadly record spatiotemporal activity, there are no tools available to conduct quantitative, statistical comparison of two groups of VSDI recordings, across the full spatiotemporal range of the acquired activity. Traditionally, VSDI analysis is conducted on a small subset of the dataset, in hand-selected regions-of-interest (ROIs). First, temporal signals are visually inspected to evaluate possible differences between groups. If differences are apparent, the investigator conducts a quantitative comparison by averaging fluorescence values over a spatial and temporal ROI in each slice, and then uses a t-test or ANOVA to determine if the perceived difference is significant.

Traditional ROI-based analysis can be sufficient if the investigator has prior knowledge of the result of the experiment and the chosen ROIs are sufficient to capture the full extent of the predicted differences in activity in the dataset. However, if the investigator's *a priori* information is incorrect or incomplete, the analysis may fail to detect true differences in activity in the dataset. A large amount of data, often the majority of the VSDI dataset, is routinely excluded from consideration when the scope of the analysis is limited to a small spatiotemporal ROI.

We developed a software toolbox to address these problems. In this report we use the toolbox to identify changes in hippocampal function in *Arx^−/+^;Dlx5/6^CIG^* mice, a transgenic model of epilepsy [8]. In mutant and control brain slices, VSDI was used to record activity evoked by stimulation of two major cortical inputs to the hippocampus, the temporoammonic pathway and the perforant pathway.

First, we used the toolbox to geometrically transform each recording from a 3D movie into a 2D image. In the 2D representation of the data, the investigator can visualize activity across the spatial and temporal scope of each recording, and data from different subjects can be visualized side by side, at once, for direct visual comparison. This new approach is in contrast to the conventional approach to analysis, where the investigator inspects temporal traces from a set of hand-selected spatial ROIs to try to comprehend what trends occur across space, or the investigator watches movies of the data to try to appreciate what trends have occurred over time in each movie.

Next, we used the toolbox to conduct statistical analysis of the data. Because the toolbox co-aligns all of the recordings to a standardized spatiotemporal map, statistical comparisons at thousands of ROIs can be conducted automatically. The result of this analysis is a 2D map that identifies significant differences in the data across spatial and temporal dimensions.

## Materials and Methods

All animal protocols were approved by the Institutional Animal Care and Use Committee at the Children's Hospital of Philadelphia. For euthanasia, mice were anesthetized with isoflurane (1–2%) and decapitated. This method is consistent with the recommendations of the American Veterinary Medical Association Guidelines on Euthanasia.

The VSDI toolbox is implemented in Matlab (version 2010b, The Mathworks, Natick, MA). All of the software described and sample data are available in **Dataset S1** and for download from Matlab Central. Detailed descriptions of the methods used to record VSDI data, methods used to generate *Arx^−/+^;Dlx5/6^CIG^* mice, and additional detail on physiological and statistical methods are available in **Supporting Information S1**. Instructions for using the software are provided in **Supporting Information S2**.

### Transformation of VSDI data from 3D to 2D

**Figure 1:**
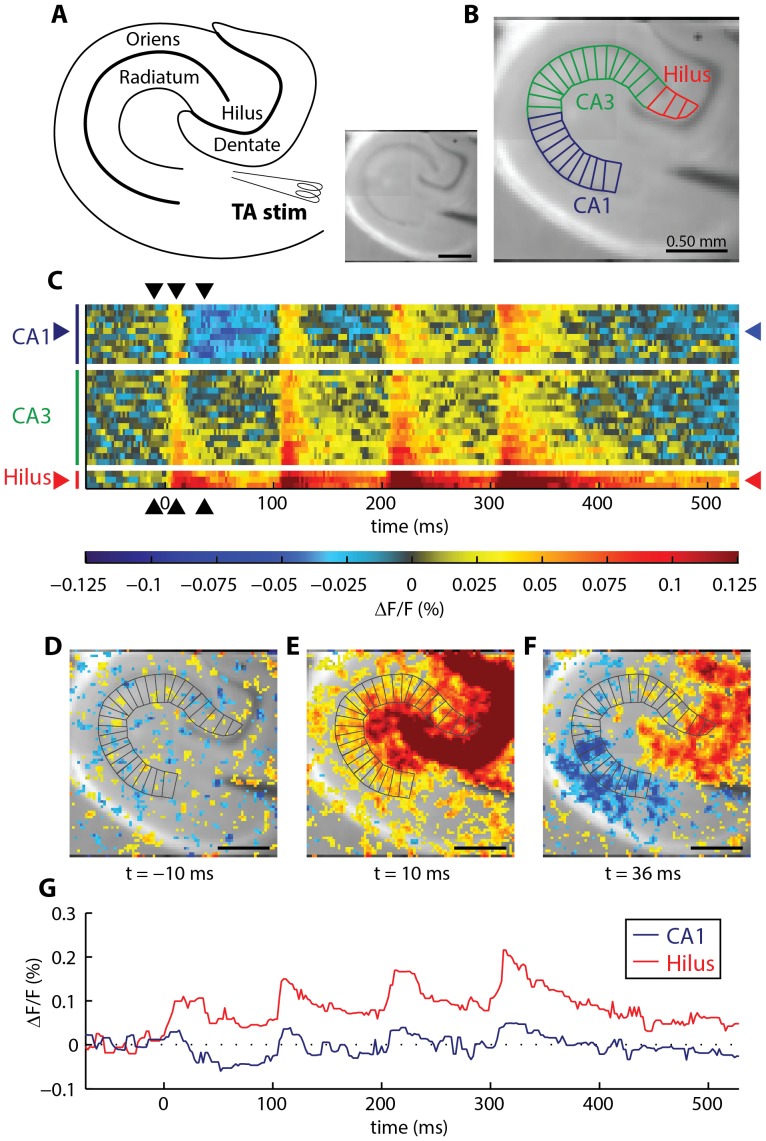
Geometric transformation of activity in a single brain slice, in stratum radiatum, evoked by temporoammonic pathway stimulation. (**A**) Schematic and VSDI camera frame showing the hippocampal anatomy. (**B**) Framework for transforming 3-D movie data (x,y,time) to 2D raster image (polygon position, time). An average temporal signal is obtained from the pixels enclosed by each polygon. (**C**) After transformation, a raster plot completely displays the spatiotemporal response in the stratum radiatum. Warmer colors indicate depolarization; cooler colors indicate hyperpolarization. Each row of the raster is a temporal trace from one polygon in **B**. From bottom to top, rows proceed from the Hilus, to CA3, to CA1. White rows indicate transitions between the anatomical regions. (**D–F**) Full VSDI camera frames, showing activity at (**D**) −10 ms (**E**) +10 ms, and (**F**) +36 ms (stimulus occurs at t = 0). For comparison, these time frames correspond to the black arrowheads that mark the columns in **C**. To aid visualization of the anatomy in **D–F**, ΔF/F values within 1.5× of the standard deviation of pre-stimulus noise were excluded from pseudocoloring (no points were excluded from pseudocoloring in the rasters). (**G**) Temporal activity at selected hilus and CA1 polygons. Spatial positions of the selected polygons are indicated with red (hilus) and blue (CA1) arrowheads in **C**.

Many of the brain slice preparations commonly used for electrophysiology (e.g., hippocampal, cerebellar, and cortical slices) are laminar. Pyramidal cell somata are arranged in a layer, with dendritic arbors fanning out in both the apical and basal directions. The image segmentation method implemented in the toolbox is designed to align with, and take advantage of, this anatomical geometry. Workflow in the toolbox begins with geometric transformation of the dataset from a 3D movie (x,y,time) to a 2D rasterized image (anatomical position, time). First, the user outlines the brain slice anatomy: in hippocampal slices (**Figure 1A**) the pyramidal cell layer, the radiatum, and the oriens are outlined. Transition points along the pyramidal cell layer are marked with hatch marks, to indicate the beginning of the pyramidal cell layer in the hilus, and the CA1–CA3 transition point (i.e., CA2). These geometric elements need only be drawn once per slice, and can be re-loaded and applied to all recordings in a given slice.

To remove jitter from hand drawn lines, the region midline (pyramidal cell layer line) was smoothed with a 99 point (∼0.216 mm arc length) averaging filter. The toolbox then automatically divides these anatomical regions into fixed-width polygonal segments (**Figure 1B**), and an average signal is calculated for each polygonal segment. Segment width is measured along the midline arc of the region (in our data, this is the pyramidal cell layer arc). Some variations may arise in the area of tissue included in each polygonal segment, as a result of this fixed-width methodology. We used a fixed-width approach because fixed-width segments correspond intuitively to the underlying anatomy of the hippocampal slice preparation: the linear arrangement of hippocampal pyramidal cell bodies can be best divided into equal populations of neurons by segmenting the cell body layer into equal length segments. A fixed-area segmentation approach could arguably be better suited to regions such as the hilus, where cells are not linearly arranged. Because the majority of the hippocampal preparation consists of linearly arranged cell bodies, we have used the fixed-width segmentation approach. In slice preparations such as this, where the region midline is smoothly curved, the difference between the resulting regions (fixed-width versus fixed-area) is expected to be very small.

The user can specify a segment width appropriate for the preparation in the settings file; segments should be wide enough to provide averaging across enough pixels to permit discrimination of signals from noise, but narrow enough that functionally distinct regions of the preparation are treated separately. In our experience a range of segment widths fit these criteria and produce qualitatively and quantitatively similar results (see *Results and Discussion*); we chose to use a segment width of 0.1 mm, which falls within this range and gives a large number of spatial sites with signals that are easily discriminated from noise. The height of each polygon is determined by the intersection of the radial segment boundary lines with the user-outlined anatomical boundaries of the preparation.

A 10 ms wide median filter is applied to each signal to improve signal/noise. The signal from each polygon is rendered as a row in a raster image (**Figure 1C**, example signals are shown in **Figure 1G**). Columns in the raster correspond to time points in the original dataset (**Figure 1D,E,F**). A detailed example of the correspondence between the untransformed, 3D dataset and the transformed 2D raster image is provided in **Movie S1**. If desired, additional structures in the slice can be geometrically transformed in the same manner. Geometric transformations of the hippocampal stratum lacunosum-moleculare and the dentate gyrus are described in **Supporting Information S2**.

### Color scale for raster plots

The toolbox uses a custom color map to display spatiotemporal activity, with warm colors representing depolarization and cool colors representing hyperpolarization. A linear ramp in color saturation and value, with gray at zero, was incorporated into the colormap (**Figure 1C**). In our experience, this color gradient allows the investigator to better resolve low amplitude signals where the signals are spatially or temporally clustered in raster plots, even when these signals would fall within the nominal noise threshold for a single pixel.

Noise thresholding is often applied to *in vitro* VSDI data to aid in the visualization of activity in a movie format [9], [10], [11]. In that approach, pseudocolor is used to render ΔF/F values that exceed the noise threshold, while the raw camera frame pixel value (grayscale) is shown at pixels with ΔF/F values below the noise threshold. We performed this type of thresholding in **Figure 1D–F** and in **Movie S1**, with the noise threshold set as 1.5 standard deviations of the baseline noise level. In movies, the advantage of the pseudocolor thresholding approach is that the anatomical boundaries of the slice are visible, at least in part, during the data review process. The disadvantage of pseudocolor thresholding is that small ΔF/F fluctuations near the thresholding level appear deceptively large, because a slightly-suprathreshold regional response will appear as a widespread pseudocolor change, while a slightly subthreshold response will register no pseudocolor change. In raster plots, noise thresholding was not used because it is not needed to understand the slice anatomy; in rasters, the slice anatomy is described by the Y axis position.

### Co-alignment of slices

The morphology of each slice is different. To permit direct comparison between slices, the toolbox converts all rasters to a standard number of spatial segments for each defined anatomical region, by interpolating rasters along the y-axis (the anatomical/spatial dimension). For the hilus, CA3, and CA1, we interpolated to give 4, 24, and 16 rows for each region, respectively. These values were chosen to be slightly larger than the number of rows in any of the individual slices studied, so that resampling would be conducted similarly in all subjects as a slight increase in sampling rate. The Matlab function resample() is used to perform linear interpolation to insert additional raster rows as necessary, so that each raster is “stretched” to be the same size. Linear interpolation was used because *in vitro* hippocampal VSDI signals are not sharply variable from segment to segment. While raster plots have been used previously to aid in VSDI data visualization [10], [12], the spatial scope of those studies was limited to small linear sub-regions of the preparation, and to our knowledge interpolation has not been previously applied to co-align VSDI brain slice data to a standardized geometric representation.

**Figure 2:**
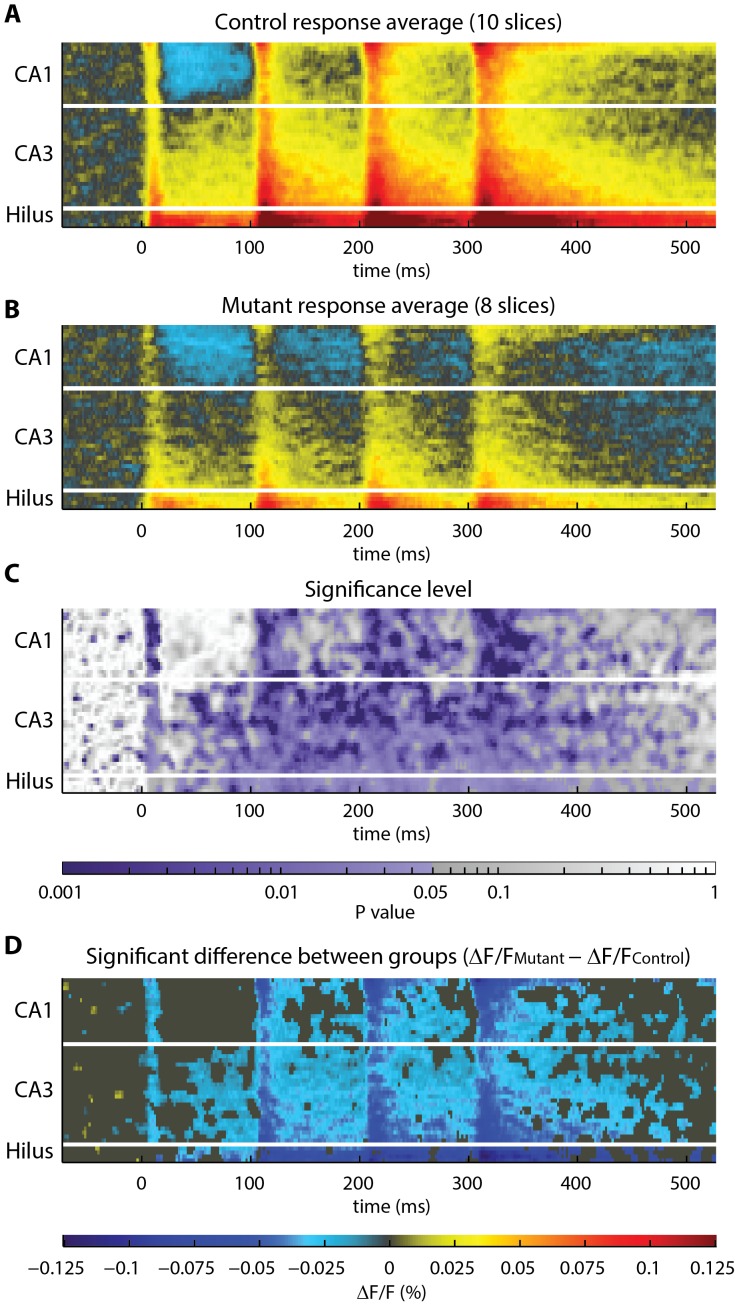
Statistical analysis of the response to temporoammonic stimulation in the stratum radiatum. (**A–B**) Rasters from multiple recordings are averaged to show overall trends in activity in the (**A**) control and (**B**) mutant groups of rasters. Visual inspection suggests that activity is different between groups. (**C**) Heatmap showing the degree of difference in activity between groups, across space and time. Statistically significant p-values (p<0.05) are shaded purple. (**D**) To obtain a spatiotemporal map of the significant difference in activity in mutant hippocampus, the control raster **A** was subtracted from the mutant raster **B**. A threshold was applied to display only sites of significant difference (p<0.05). Significant differences were registered at 7499 of 13244 sites (57%). Color scale is the same in **A**, **B**, and **D**.

After converting each raster to standard dimensions, the rasters can be averaged together to visually compare activity between groups of slices at co-aligned spatiotemporal coordinates (**Figure 2**). It is straightforward to conduct statistical analyses using this representation of the data. Additionally, the toolbox also contains utilities to make other types of measurements by taking advantage of the polygonal representation of the slice geometry: the toolbox measures the velocity of the spread of electrical activity and tests for anatomical differences between groups, as described in *“Activation velocity measurement”* and *“Comparison of slice anatomy”*, below.

### Statistical analysis

Statistical comparison between groups is performed at all spatiotemporal sites with a permutation test. This is a non-parametric test that resamples the data to generate a null distribution describing the variability in the data at each site. Significant differences are registered at sites where the experimental versus control groupings explain the variability in the data. The toolbox renders the result of the permutation test as a heatmap of p-values for all sites (**Figure 2C**). To aid in visualization of the magnitude of the activity difference, the control raster is subtracted from the mutant raster, and only the ΔF/F differences at sites with p<0.05 are plotted (**Figure 2D**).

### Influence of segment width on statistical analysis output

To determine if the toolbox results were dependent upon our choice of a fixed polygonal segment width of 0.1 mm, we evaluated the possible influence of the width of each polygonal segment on the output of the statistical analysis by repeating the analysis on rasters generated from polygonal geometries with different segment widths. This procedure was conducted on two datasets: rasters of temporoammonic pathway-evoked stratum radiatum activity, and rasters of perforant pathway-evoked stratum radiatum activity. Raster plots were generated from polygonal geometries with each of the following 15 segment widths (in mm): 0.020, 0.040, 0.060, 0.080, 0.100, 0.120, 0.140, 0.160, 0.180, 0.200, 0.220, 0.240, 0.260, 0.280, and 0.300. We conducted permutation tests to compare mutant to experimental data for each of these 15 sets of rasters.

### Activation velocity measurement

In control slices, a single stimulus was delivered to the Schaffer collateral axons and evoked activity was recorded with VSDI (**Figure S1A,B**). Signals resulting from this stimulation paradigm consist of a combination of both presynaptic fluorescence from the Schaffer collateral axons, and postsynaptic fluorescence from CA1 dendritic arbors. Accordingly, the velocity of the spread of activity relates to latencies in propagation in the presynaptic and postsynaptic cells.

Velocity was measured using a least-squares linear fit to a position versus time plot, as previously described [13]. Briefly, in each row of the raster, the activation time was measured as the local maximum in the derivative of the signal. The slope of the linear fit to the activation times for all signals yielded the velocity of the spread of activity (**Figure S1C**). Distances between measurements sites were taken as the Euclidean distance between the geometric center of each polygonal measurement site. To maintain real-world units of distance in activation velocity measurements, we did not alter the number of rows in raster plots, as we did to compare spatiotemporal activity between groups (as described above in *Co-alignment of slices*). Activation velocity was 0.13±0.03 m/s (n = 10 recordings, **Figure S1D**).

### Comparison of slice anatomy

Mutations in genes that alter normal brain development often produce abnormalities in brain anatomy. Anatomical abnormalities have been observed in mouse models of some, but not all, mutations in the *Arx* gene [8], [14], [15]. To determine if the *Arx^−/+^;Dlx5/6^CIG^* mutation produces anatomical abnormalities in the adult mouse hippocampus, we compared the anatomy of mutant slices to the anatomy of control slices. Using the geometric parameters of each slice, it was straightforward to compare the physical dimensions of anatomical features in mutant and control groups (**Figure S2**). The length of the pyramidal cell layer and the area of the raster geometry were assessed for each slice. Note that the polygonal segments were not altered (“stretched”) prior to this analysis; comparisons were conducted using real-world lengths (mm) and areas (mm^2^) of anatomical regions. The length of the pyramidal cell layer was not significantly different between groups (p = 0.26). The area of the hippocampus was 11% larger in the mutant (p = 0.03).

### Signal-to-noise analysis

To determine if the signal-to-noise ratio was affected by anatomy-guided image segmentation, regionally averaged signals were compared between pixel clusters that were selected in two ways: (1) anatomically-guided wedge-shaped polygonal segments, and (2) circular regions with the same center. We hypothesized that signals from a same-sized pixel cluster would have comparable signal/noise ratio in both cases. Signal-to-noise ratio was computed as the ratio of the peak ΔF/F value during the excitatory postsynaptic potential, to the root-mean-squared noise level during the pre-stimulus interval [16]. We found that signal-to-noise ratios were equivalent between the two methods (**Figure S3**).

## Results and Discussion

To explore the utility of the VSDI toolbox, we first tested for a difference in temporoammonic pathway-evoked activity in the stratum radiatum in mutant slices compared to control slices. Upon inspection of the difference in activity between mutant and control groups, several features are immediately apparent (**Figure 2C,D**). The decreased response immediately following the first stimulus indicates that glutamatergic transmission is depressed in CA1 of the mutant. Activity immediately following the first stimulus is also decreased in area CA3, though the activity observed in CA3 is likely a result of simultaneous stimulation of perforant pathway fibers, as CA3 has minimal innervation from the temporoammonic pathway. By contrast, the initial inhibitory event in CA1 (blue pixels in **Figure 2A,B**) is similar between groups, suggesting that the excitatory/inhibitory balance of the mutant network is shifted toward inhibition. The widespread depression in the response to stimulus numbers 2–4 in the mutant could be a result of either depression of excitatory synaptic responses or disinhibition of inhibitory synapses. Further studies could differentiate these possibilities.

We also used the toolbox to analyze activity in the stratum oriens evoked by temporoammonic pathway stimulation. **Figure S4** shows the polygonal geometry used to segment the stratum oriens, and the corresponding raster plot of stratum oriens activity for a single VSDI recording. **Figure S5** shows the result of statistical comparison of temporoammonic pathway-evoked stratum oriens activity, between mutant and control groups of slices. Similar to the stratum radiatum, temporoammonic pathway-evoked activity in the stratum oriens was depressed in mutant mice.

**Figure 3:**
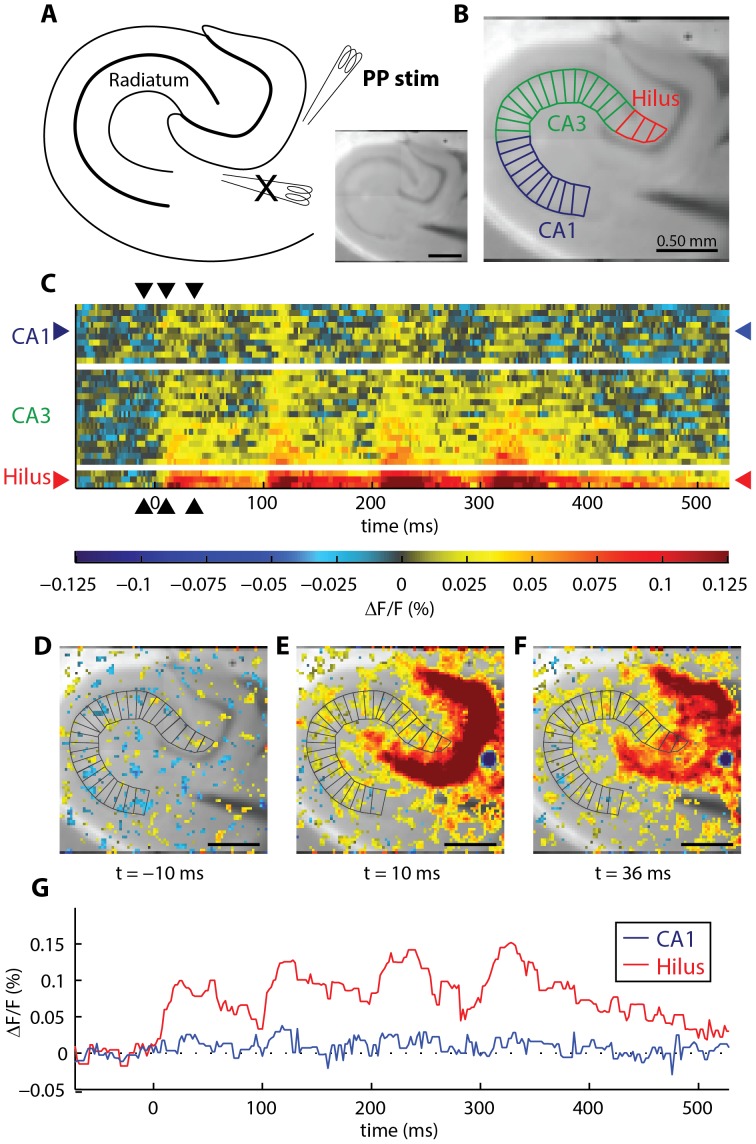
Geometric transformation of activity in stratum radiatum, evoked by perforant pathway stimulation. (**A**) Schematic and VSDI camera frame showing the hippocampal anatomy and the position of the stimulating electrode. Two electrodes were placed in the slice, but only the electrode labeled “PP stim” (visible at the right edge of the raw image) was used to deliver the stimulus. The remaining electrode, marked with an “X” in the schematic, was unplugged during this recording. (**B**) Polygonal geometry for transforming data to 2D. A temporal signal is obtained from each polygon. (**C**) After transformation, a raster plot completely displays the spatiotemporal response in the stratum radiatum. Warmer colors indicate depolarization; cooler colors indicate hyperpolarization. Each row of the raster is a temporal trace from one polygon in **B**. From bottom to top, rows proceed from the Hilus, to CA3, to CA1. White rows indicate transitions between the anatomical regions. (**D–F**) Full VSDI camera frames, showing activity at (**D**) −10 ms (**E**) +10 ms, and (**F**) +36 ms (stimulus occurs at t = 0). For comparison, these frames correspond to the black arrowheads that mark the columns in **c**. To aid visualization of the anatomy in panels **D–F**, ΔF/F values within 1.5× of the standard deviation of pre-stimulus noise were excluded. (**G**) Temporal activity at selected hilus and CA1 sites. Spatial positions of these signals are indicated with red (hilus) and blue (CA1) arrowheads in **C**.

**Figure 4:**
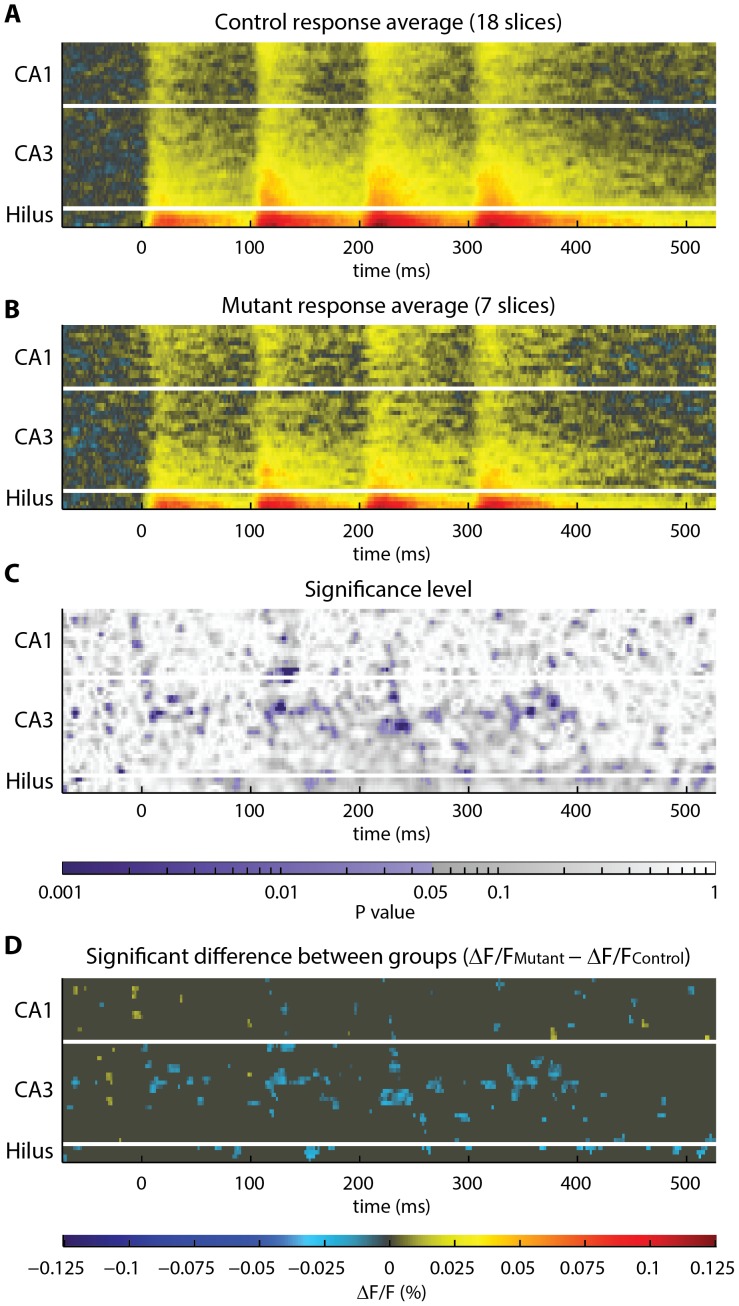
Statistical analysis of the response in stratum radiatum to perforant pathway stimulation. This analysis was conducted in the same manner as shown in **Figure 2**. (**A–B**) Visual inspection of the averaged (**A**) control and (**B**) mutant rasters suggests that activity is similar in both groups. (**C**) Heatmap showing the degree of difference in activity between groups, across space and time. Statistically significant p-values (p<0.05) are shaded purple. (**D**) To obtain a spatiotemporal map of the significant difference in activity in mutant hippocampus, the control raster **A** was subtracted from the mutant raster **B**. A threshold was applied to display only sites of significant difference (p<0.05). Significant differences were registered at 669 of 13244 sites (5%). For α = 0.05, we expect 5% of sites to be identified as significantly different by chance. Therefore, these data indicate that perforant pathway evoked activity in the stratum radiatum is not significantly different between groups. Color scale is the same in **A**, **B**, and **D**.

To investigate the extensibility of the toolbox to different stimulation protocols, we used the toolbox to analyze activity evoked by perforant pathway stimulation in the stratum radiatum and stratum oriens. The polygonal geometry used to segment the stratum radiatum in recordings of perforant pathway-evoked activity is shown in **Figure 3**. The result of the statistical analysis of perforant pathway-evoked stratum radiatum activity, in mutant versus control slices, is shown in **Figure 4**. The polygonal geometry used to segment the stratum oriens in recordings of perforant pathway-evoked activity is shown in **Figure S6**. The result of the statistical analysis of perforant pathway-evoked stratum oriens activity, in mutant versus control slices, is shown in **Figure S7**. The number of sites identified as significantly different in recordings of perforant pathway evoked activity (5.4% in stratum radiatum, 5.1% in stratum oriens) is roughly equivalent to the number of sites that would be expected to be identified as significantly different by chance (5%, for α = 0.05). Therefore, we observed no significant differences in perforant pathway activity in our data, indicating that the decrease in excitability that we observed in the temporoammonic pathway is not universal to all circuits in the mutant.

**Figure 5:**
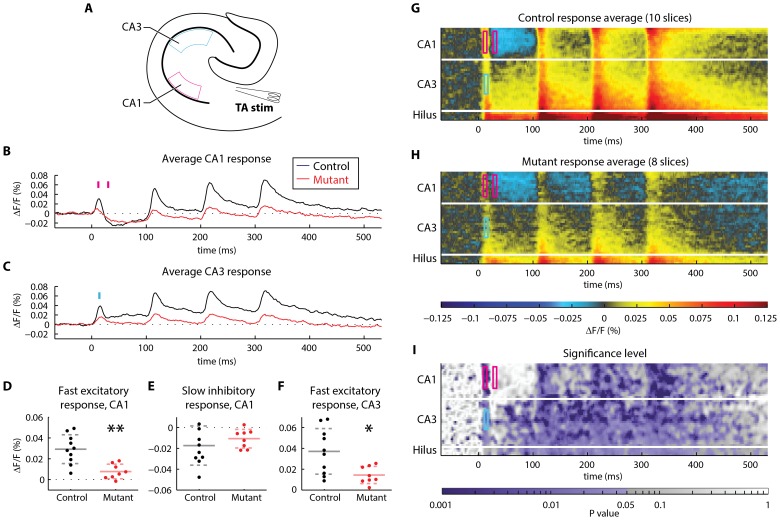
Spatiotemporal analysis, compared to conventional analysis, of the response to temporoammonic pathway stimulation in mutant (n = 8) and control (n = 10) slices. (Same data as in **Figure 2**.) (**A**) In the first step of conventional VSDI analysis, a temporal signal is obtained for a region of interest (ROI) by averaging the data across a cluster of pixels and over a time interval of interest. Here, we have selected two regions: CA1 stratum radiatum and CA3 stratum radiatum. (**B**) The average response in the CA1 stratum radiatum ROI to four, 10 Hz stimuli delivered to the temporoammonic pathway in mutant (red) and control (black) slices in CA1 stratum radiatum. Conventional analysis proceeds by identifying time intervals of interest in these data. Here, we have chosen time intervals corresponding to the fast excitatory postsynaptic potential (EPSP) and the slow inhibitory hyperpolarization that follows the stimulus. Both of these time intervals are marked with magenta bars and these intervals are 6 ms long (3 camera frames at 500 frames per second). (**C**) The average response in the CA3 stratum radiatum ROI, in the same recordings of mutant and control slices. We have chosen to analyze the time interval corresponding to the fast EPSP for analysis in CA3; this 6 ms-long interval is marked with a cyan bar. (**D–F**) Traditional (ROI-based) statistical comparison of voltage-sensitive fluorescence in mutant and control slices in (**D**) CA1 during the fast EPSP, (**E**) CA1 during the slow inhibitory response, and (**F**) CA3 during the fast EPSP. Significant differences were observed in the fast EPSP in CA1 and in the fast EPSP in CA3 (t-test; ** P<0.01, * P<0.05; solid and dashed lines indicate mean±standard deviation). No significant difference was observed in the slow inhibitory response in CA1. (**G–H**) Rasters of average activity in (**G**) control and (**H**) mutant slices. Visual inspection suggests that activity is qualitatively different between groups across many sites. (**I**) Heatmap showing sites of significantly different spatiotemporal activity that were identified by the permutation test. The spatiotemporal sites that were analyzed using conventional VSDI analysis (described in **A–F**) are outlined in CA1 (magenta) and CA3 (cyan). The spatial (vertical) and temporal (horizontal) dimensions of these boxes match the spatial and temporal extent of the conventional ROI analyses performed in panels **A–F**. All boxes are 6 ms (3 samples) wide.

After identifying many sites of significant difference in the temporoammonic pathway dataset, we evaluated the sensitivity of the toolbox by comparing the result of the permutation test to the p-values obtained using the traditional VSDI statistical analysis approach (**Figure 5**). We conducted traditional ROI analyses at spatiotemporal sites where the permutation test showed a preponderance of highly significant differences (CA1, immediately following the stimulus), no significant differences (CA1, 20 ms after the stimulus), and moderately significant differences (CA3, immediately following the stimulus). In these ROIs, p-values obtained using a T-test were 0.0011, 0.3746, and 0.0142, respectively (**Figure 5D–F**). In the same ROIs, the permutation test yielded average p-values of 0.0047, 0.4395, and 0.0287, respectively (**Figure 5I**). This analysis indicates that the automatic comparisons performed by the toolbox yield a similar level of sensitivity as traditional VSDI analysis methods.

As the number of statistical comparisons is increased, the likelihood that one of those comparisons will erroneously register a significant difference is also increased. When multiple statistical comparisons are conducted, it is important to consider how the multiplicity of hypothesis testing influences the result. Multiple comparisons corrections, such as the Benjamini-Hochberg or Bonferroni procedures [17], are inadequate in the present context, because spatial and temporal correlations exist in the data such that all comparisons (in our case, comparisons at 13,244 sites) are not truly independent. Accordingly, in this setting, a more suitable approach is to choose a significance threshold that yields an acceptable false positive detection rate, and to estimate the proportion of sites expected to be falsely positive when interpreting the toolbox output.

**Figure 6:**
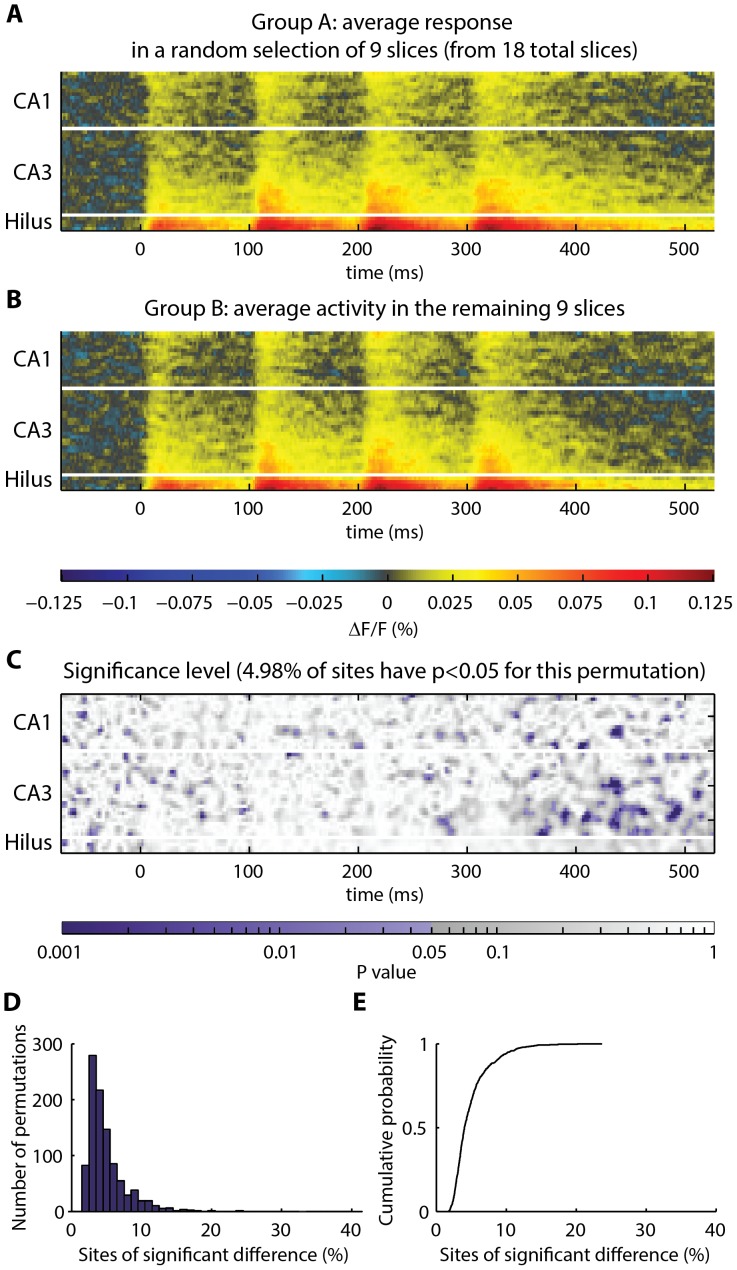
Frequency of sites identified as significantly different in control data: response in stratum radiatum following perforant pathway stimulation. (**A–B**) A total of 18 control slices were shuffled into two random groups, “Group A” and “Group B”. The average of each group is shown. (**C**) Heatmap, showing p-values obtained by comparing Group A to Group B at each spatiotemporal site. P-values less than 0.05 are colored purple. For the random Groups A and B, significant differences were registered at 4.98% of sites. (**D–E**) Histogram and cumulative probability distribution, showing the number of positive sites obtained from permutation test comparison of 1000 random groupings of the slices. Under the null hypothesis, the theoretical rate of observation of positive sites is predicted to be 5% for α = 0.05. In 1000 permutations of the actual data, 4.90% of sites were registered as significantly different.

**Figure 7:**
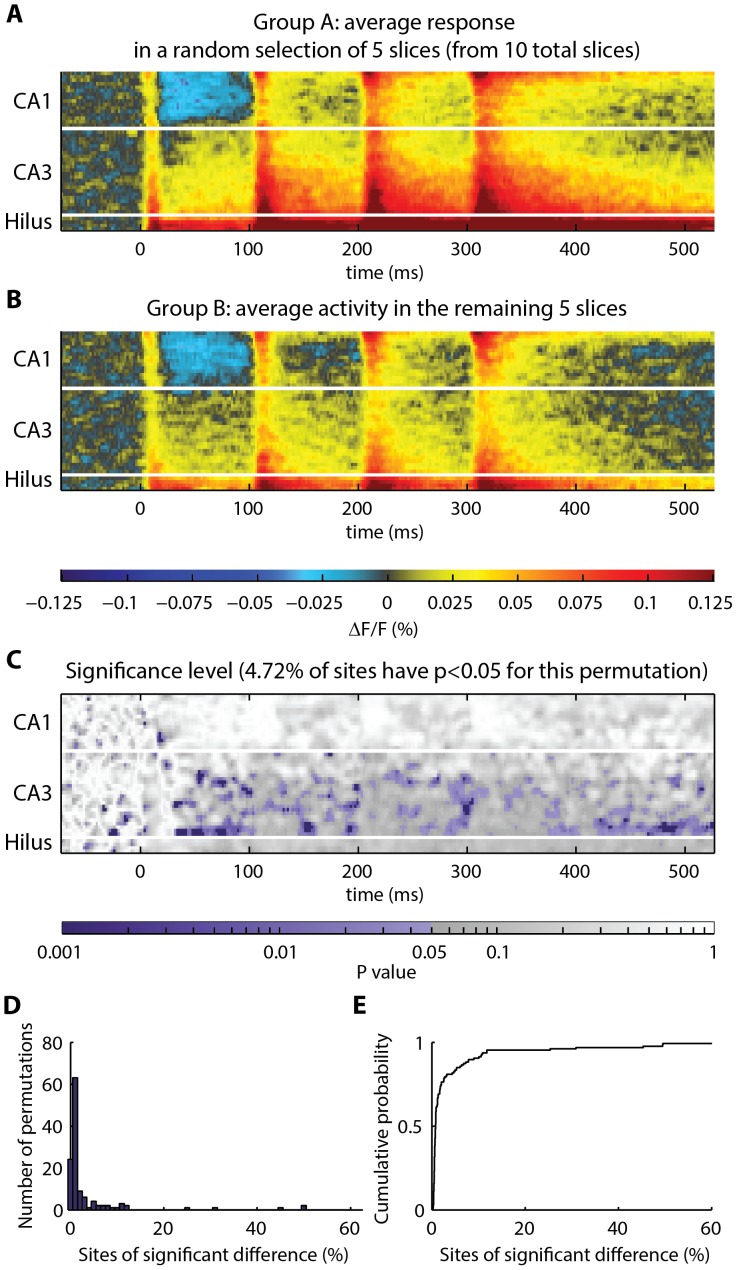
Frequency of sites identified as significantly different in control data: response in stratum radiatum following temporoammonic pathway stimulation. (**A–B**) A total of 10 slices were shuffled and divided into two groups, “Group A” and “Group B”. The average of each group is shown. (**C**) Heatmap, showing p-values obtained by comparing Group A to Group B at each spatiotemporal site. P-values less than 0.05 are colored purple. For the random Groups A and B, significant differences were registered at 4.72% of sites. (**D–E**) Histogram and cumulative probability distribution, showing the number of positive sites obtained from permutation test comparison of all possible groupings of the data into 2 groups of 5 (126 unique combinations). Under the null hypothesis, the theoretical rate of observation of positive sites is predicted to be 5% for α = 0.05. In 126 permutations of the actual data, 3.97% of sites were registered as significantly different.

A direct approach to multiple comparisons consists of selecting a significance threshold for each site that achieves an acceptably low rate of false positive detections. This is achieved in our setting by selecting an appropriate α-level. A practical estimate of the number of sites of true significance in the toolbox output is the difference between the number of sites discovered in the real comparison versus the number of false-positive sites that are routinely discovered by chance under a null scenario, defined through permutations, such that all null hypotheses hold. To verify that the toolbox would identify sites as “significantly different” by chance at a rate consistent with the α-level, control slices were divided into two groups and the permutation test was performed. The control slices are expected to be physiologically similar to each other. Therefore, for α = 0.05, it is expected that approximately 5% of sites will be identified as significantly different by chance. Consistent with this expectation, the toolbox registered significance at 4.90% of sites in the perforant pathway dataset (**Figure 6**). The toolbox registered significance at 3.97% of sites in the temporoammonic pathway dataset (**Figure 7**). If a different α-level is desired, this can be adjusted in the toolbox settings.

**Figure 8:**
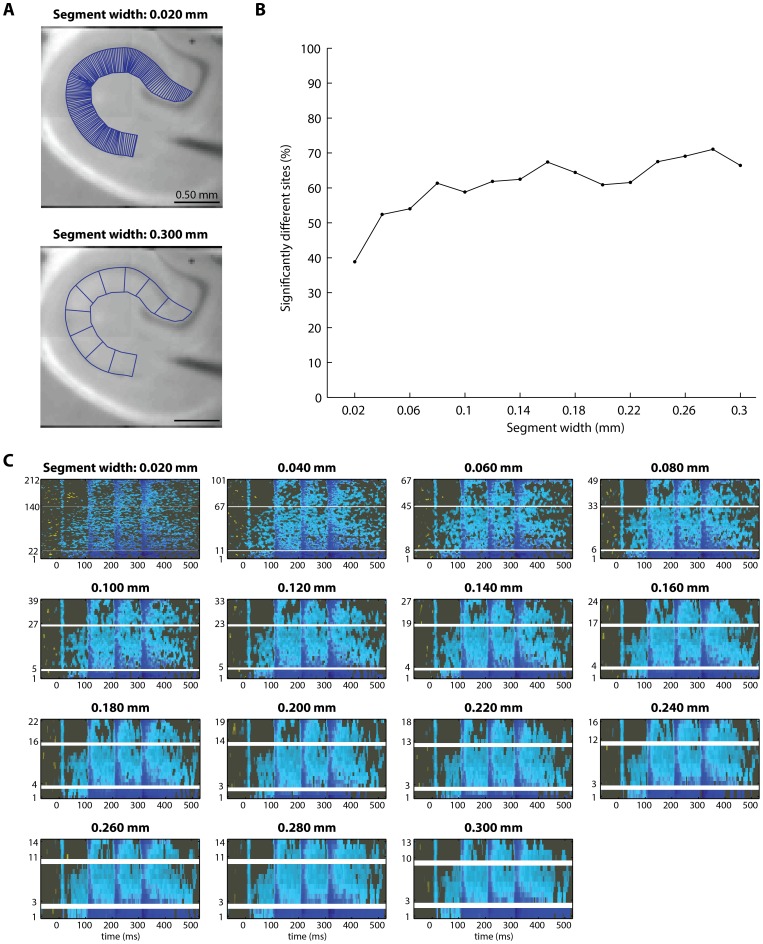
In temporoammonic pathway evoked activity in the stratum radiatum, the statistical analysis output is similar regardless of the chosen width of polygonal segments for image segmentation. To determine if the percentage of spatiotemporal sites identified as significantly different was a function of the width of polygonal segments used in image segmentation, the image segmentation procedure was repeated fifteen times, to generate rasters from polygonal geometries with segment widths ranging from 0.020 mm to 0.300 mm, in 0.020 mm increments. Permutation testing was conducted to compare experimental rasters to control rasters that were generated from geometries with each of the fifteen segment widths, to determine if segment width influenced the output of the permutation test. (**A**) Polygonal geometries are shown for the narrowest (0.020 mm) and the widest (0.300 mm) segment widths tested. (**B**) The percentage of sites identified as significantly different for each of the fifteen segment-width iterations. The percentage of sites identified as significantly different is similar across most segment widths. One notable exception occurs at segment width = 0.020 mm, where fewer sites of significant difference were identified. This is probably because the segment width (0.020 mm) is narrower than the pixel width (0.025 mm), so these segments each include few pixels and therefore the resulting signals from these narrow segments have poor signal-to-noise ratio. (**C**) The significant difference in activity, identified by permutation testing, for each of the fifteen segment widths tested (each panel corresponds to one tested segment width). The difference in activity is shown in the same format as in **Figure 1D**: (mutant activity – control activity), thresholded to show only sites of significant difference at α = 0.05. Qualitatively, the output of the permutation test is similar enough that the same conclusions about the underlying physiology could be drawn from consideration of any of the panels.

**Figure 9:**
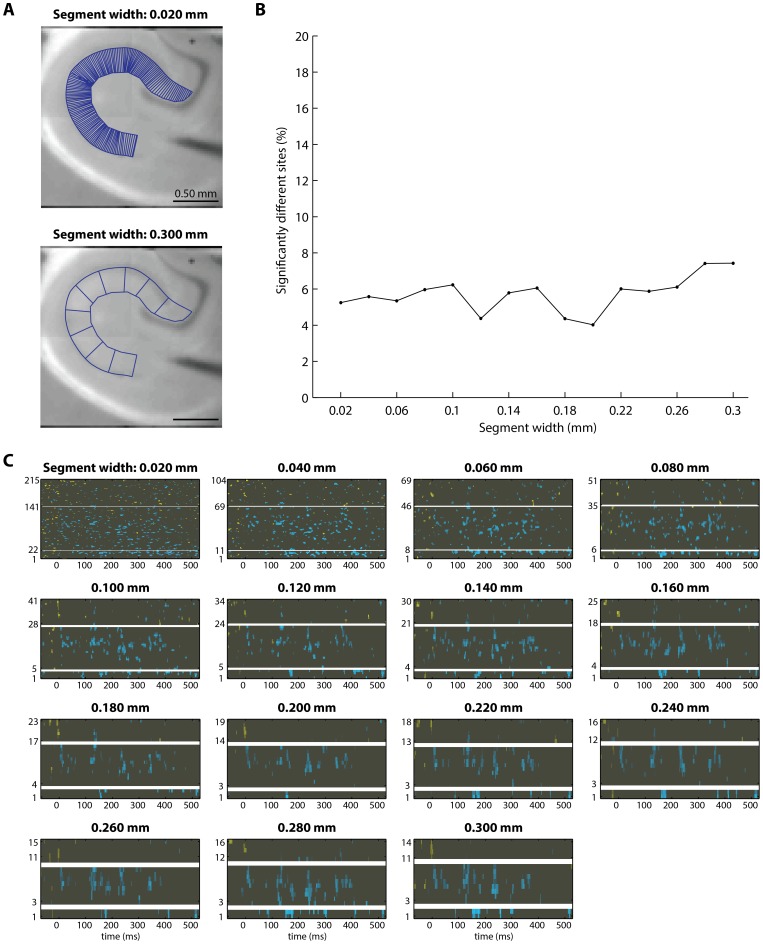
In perforant pathway evoked activity in the stratum radiatum, the statistical analysis output is similar regardless of the chosen width of polygonal segments for image segmentation. (This is the same test shown in **Figure 8**, applied to perforant pathway VSDI data.) To determine if the percentage of spatiotemporal sites identified as significantly different was a function of the width of polygonal segments used in image segmentation, the image segmentation procedure was repeated 15 times, to generate rasters from polygonal geometries with segment widths ranging from 0.020 mm to 0.300 mm, in 0.020 mm increments. Permutation testing was conducted to compare experimental rasters to control rasters that were generated from geometries with each of the fifteen segment widths, to determine if segment width influenced the output of the permutation test. (**A**) Polygonal geometries are shown for the narrowest (0.020 mm) and the widest (0.300 mm) segment widths tested. (**B**) The percentage of sites identified as significantly different for each of the fifteen segment-width iterations. The percentage of sites identified as significantly different is similar across most segment widths. For all segment widths, the percentage of sites identified as significantly different is similar to the percentage of sites expected to be identified as significantly different by chance (∼5% of sites at α = 0.05). (**C**) The significant difference in activity, identified by permutation testing, for each of the fifteen segment widths tested (each panel corresponds to one tested segment width). The difference in activity is shown in the same format as in **Figure 1D**: (mutant activity – control activity), thresholded to show only sites of significant difference at α = 0.05. Qualitatively, the output of the permutation test is similar enough that the same conclusions about the underlying physiology could be drawn from consideration of any of the panels.

To evaluate the influence of segment width on the statistical output, we conducted permutation testing on raster datasets generated from polygonal geometries of 15 different segment widths. For all fifteen segment-widths tested, 61.2±8.1% sites were identified as significantly different in rasters of temporoammonic activity (**Figure 8**) and 5.7±1.0% of sites were identified as significantly different in rasters of perforant pathway activity (**Figure 9**). The percentage of sites identified as significantly different increased slightly as segment width increased. This is probably due to higher signal-to-noise ratios in larger segments, where more pixels are averaged together to generate each raster signal. In support of this idea, the fewest sites of significant difference were identified when the segment width was fixed at 0.020 mm, which is narrower than the width of a single camera pixel (0.025 mm). The map of significant differences was not qualitatively different when different segment widths were used to analyze temporoammonic pathway activity (**Figure 8C**). Similarly, the map of significant differences was not qualitatively different when different segment widths were used to analyze perforant pathway activity (**Figure 9C**). These results indicate that the conclusions that can be drawn from inspection of the output of the permutation test are minimally affected by the user's choice of segment width.

The utility of VSDI as an assay to identify network pathophysiology has been limited by the lack of a standardized, unbiased framework to visualize and interpret the full spatiotemporal extent of the data. The VSDI toolbox solves this problem for VSDI studies in hippocampus, and can be easily extended to other brain regions; stimulation paradigms, such as optogenetics; and imaging methods, such as epifluorescent calcium imaging. The toolbox makes available a core set of utilities for VSDI data visualization, normalization of data from morphologically different slices, and direct statistical comparison of spatiotemporal activity. Permutation testing, as implemented here, is increasingly used for the analysis of large datasets, such as DNA microarrays and MRI data [18], [19], but has not been previously applied to VSDI. This approach significantly enhanced our ability to detect spatiotemporal differences in activity across the hippocampus, with minimal sampling bias. Because the permutation test makes it possible to statistically compare activity at thousands of spatiotemporal sites at once, the likelihood is greatly reduced that sites of significant difference will be overlooked in the analysis. Overall, we find that this toolbox permits much more complete analysis of VSDI datasets, and we believe the availability of these tools will facilitate widespread application of VSDI to the study of functional disorders of the brain.

## Supporting Information

Figure S1 **Activation velocity measurement.** (**A**) Schematic and raw camera image showing a slice with a stimulating electrode positioned to stimulate the Schaffer collateral axons (two electrodes are visible in the image, but stimuli were only delivered through the one electrode in the stratum radiatum, labeled “SC stim”). (**B**) VSDI data from the stratum radiatum were segmented with the polygonal geometry shown. Black polygonal segments indicate the CA1 segments used for velocity measurements. (**C**) Raster showing spatiotemporal activity evoked by Schaffer collateral stimulation. The stimulus was delivered at t = 0 ms. Rows, from the bottom to the top, correspond to segments of the hippocampal geometry, from the hilus to CA3 and finally to CA1, as shown in **B**. For each row, the activation time was computed as the peak in the first derivative of the optical signal. Dots indicate activation times in each row. (**D**) Velocity measurement. For each CA1 site, the activation velocity in stratum radiatum was computed as the slope of a linear regression of distance versus activation time. The Y axis (distance) was computed as the distance between the centroids of each polygonal segment; centroid positions are indicated with blue dots in **A**. (**E**) Average activation velocity. Each point represents one activation velocity measurement. Solid and dashed gray lines show mean activation velocity and standard deviation, respectively. In all recordings, activation velocity was 0.13±0.03 m/s (n = 10).
(TIF)

Figure S2 **Anatomical comparison using slice geometries.** The VSDI toolbox parameterizes the geometry of the slice with a series of polygons to transform the VSDI dataset into a 2D raster plot. Slice anatomy can be quantitatively compared between groups using these polygons. (**A**) Schematic of analyzed anatomical features. Gray shading indicates the region of the hippocampus parameterized by the toolbox (“Raster region”). The pyramidal cell layer arc is indicated with as a heavy black line. Note that anatomical comparisons were conducted using the original, “un-stretched” polygonal geometries, so that real world units of length (mm) and area (mm^2^) are preserved. (**B**) The length of the pyramidal cell layer was not significantly different between control and mutant slices (p = 0.13). (**C**) The raster area of the hippocampus was significantly different between control and mutant slices (p = 0.03; t-test; n = 19 control and 10 mutant slices).
(TIF)

Figure S3 **Signal-to-noise analysis.** (**A**) Temporal VSDI signals were obtained from the 3D dataset using anatomically guided image segmentation (black lines), and from conventional, circular image segmentation (blue circles). Each circle has the same area and the same center of mass as its co-localized anatomically guided segment. (**B**) Temporal signals, obtained by spatially averaging across the area defined by a single anatomical segment (black signal) or circle (blue signal). These signals are from the segment and circle indicated with arrows in panel **A**. Signal power was measured as the average ΔF/F value during the peak of the excitatory postsynaptic potential (red time interval). Signals were compared to the RMS noise level during the pre-stimulus interval. The signal-to-noise ratio (SNR) was similar when spatial averaging was conducted using either anatomically guided or circular spatial averaging methods (SNR_anatomical_ = 28.5±12.4, SNR_circle_ = 29.5±13.4; n = 120 regions, paired t-test, P = NS.) This indicates that there is no significant change in temporal resolution when signals are obtained using the new image segmentation method, compared to conventional spatial averaging.
(TIF)

Figure S4 **Geometric transformation of activity in stratum oriens, evoked by temporoammonic pathway stimulation.** Data are from the same recording as shown in **Figure 1**. (**A**) Schematic and VSDI camera frame showing the hippocampal anatomy and the position of the stimulating electrode. (**B**) Polygonal geometry for transforming data to 2D. A temporal signal is obtained from each polygon. (**C**) After transformation, a raster plot completely displays the spatiotemporal response in the stratum oriens. Warmer colors indicate depolarization; cooler colors indicate hyperpolarization. Each row of the raster is a temporal trace from one polygon in **B**. From bottom to top, rows proceed from the Hilus, to CA3, to CA1. White rows indicate transitions between the anatomical regions. (**D–F**) Full VSDI camera frames, showing activity at (**D**) −10 ms (**E**) +10 ms, and (**F**) +36 ms (stimulus occurs at t = 0). For comparison, these frames correspond to the black arrowheads that mark the columns in **C**. To aid visualization of the anatomy in panels **D–F**, ΔF/F values within 1.5× of the standard deviation of pre-stimulus noise were excluded. (**G**) Temporal activity at selected hilus and CA1 sites. Spatial positions of these signals are indicated with red (hilus) and blue (CA1) arrowheads in **C**.
(TIF)

Figure S5 **Statistical analysis of the response to temporoammonic stimulation in the stratum oriens.** This analysis was conducted in the same manner as shown for stratum radiatum in **Figure 2**. (**A–B**) Visual inspection of the averaged (**A**) control and (**B**) mutant rasters suggests that activity is different between groups. (**C**) Heatmap showing the degree of difference in activity between groups, across space and time. Statistically significant p-values (p<0.05) are shaded purple. The differences seen here in stratum oriens are qualitatively similar to the differences observed in **Figure 2**. (**D**) To obtain a spatiotemporal map of the significant difference in activity in mutant hippocampus, the control raster **A** was subtracted from the mutant raster **B**. A threshold was applied to display only sites of significant difference (p<0.05). Significant differences were registered at 6345 of 13244 sites (48%). Color scale is the same in panels **A**, **B**, and **D**.
(TIF)

Figure S6 **Geometric transformation of activity in stratum oriens, evoked by perforant pathway stimulation.** Data are from the same recording as shown in **Figure 3**. (**A**) Schematic and VSDI camera frame showing the hippocampal anatomy and the position of the stimulating electrode. Two electrodes were placed in the slice, but only the electrode labeled “PP stim” was used to deliver the stimulus. The remaining electrode, marked with an “X” in the schematic, was unplugged during this recording. (**B**) Polygonal geometry for transforming data to 2D. A temporal signal is obtained from each polygon. (**C**) After transformation, a raster plot completely displays the spatiotemporal response in the stratum oriens. Warmer colors indicate depolarization; cooler colors indicate hyperpolarization. Each row of the raster is a temporal trace from one polygon in **B**. From bottom to top, rows proceed from the Hilus, to CA3, to CA1. White rows indicate transitions between the anatomical regions. (**D–F**) Full VSDI camera frames, showing activity at (**D**) −10 ms (**E**) +10 ms, and (**F**) +36 ms (stimulus occurs at t = 0). For comparison, these frames correspond to the black arrowheads that mark the columns in **C**. To aid visualization of the anatomy in panels **D–F**, ΔF/F values within 1.5× of the standard deviation of pre-stimulus noise were excluded. (**G**) Temporal activity at selected hilus and CA1 sites. Spatial positions of these signals are indicated with red (hilus) and blue (CA1) arrowheads in **C**.
(TIF)

Figure S7 **Statistical analysis of the response in stratum oriens to perforant pathway stimulation.** This analysis was conducted in the same manner as shown in **Figure 2**. (**A–B**) Visual inspection of the averaged (**A**) control and (**B**) mutant rasters suggests that activity is similar in both groups. (**C**) Heatmap showing the degree of difference in activity between groups, across space and time. Statistically significant p-values (p<0.05) are shaded purple. (**D**) To obtain a spatiotemporal map of the significant difference in activity in mutant hippocampus, the control raster **A** was subtracted from the mutant raster **B**. A threshold was applied to display only sites of significant difference (p<0.05). Significant differences were registered at 717 of 13244 sites (5.4%). Color scale is the same in **A**, **B**, and **D**.
(TIF)

Dataset S1 **VSDI toolbox software.**
(ZIP)

Supporting Information S1 **Supplemental methods section.**
(DOCX)

Supporting Information S2 **Instructions for using the VSDI toolbox software.**
(DOCX)

Movie S1 **Geometric transformation of VSDI data in the stratum radiatum.** (**A**) The original 3D VSDI dataset, in pseudocolor. Warmer colors represent depolarization and cooler colors represent hyperpolarization. Black lines indicate the polygonal geometry used for geometric transformation. Anatomical transitions between the hilus and CA3, and between CA3 and CA1, are indicated with white lines. (**B**) For each video frame, and for each polygonal segment, the fluorescence values are averaged to obtain a single fluorescence value for that segment at that time point. These average ΔF/F fluorescence values are rendered in pseudocolor for each polygonal segment. (**C**) The raster plot is assembled by appending one column of averaged fluorescence values to the raster plot for each movie frame. For each movie frame, the pseudocolor values shown in the polygonal segments of panel **B** correspond exactly to the values in the rightmost column of the raster plot. This video shows the same recording that is shown in **Figure 1**.
(AVI)
