# Supplementary figures and images for: A Toolbox for Spatiotemporal Analysis of Voltage-Sensitive Dye Imaging Data in Brain Slices
Source: PLoS One. 2014 Sep 26;9(9):e108686. doi: 10.1371/journal.pone.0108686 (PMC4178182; doi:10.1371/journal.pone.0108686)

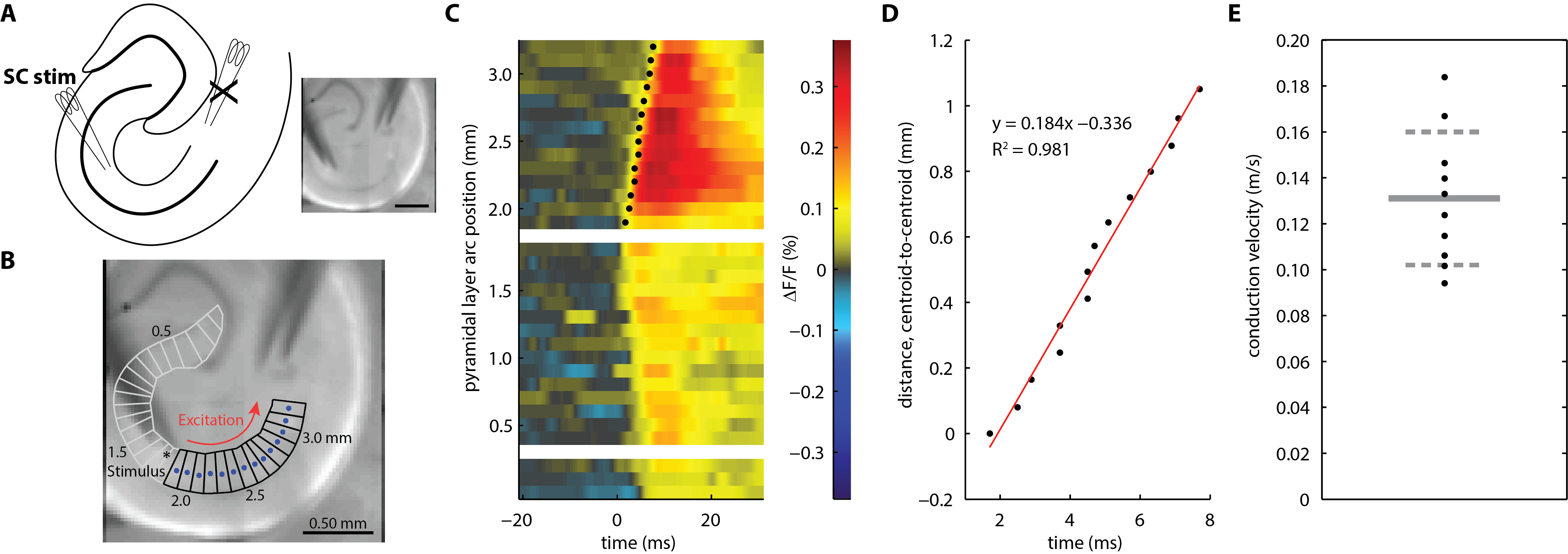

Supplement: Figure S1 — Activation velocity measurement. (A) Schematic and raw camera image showing a slice with a stimulating electrode positioned to stimulate the Schaffer collateral axons (two electrodes are visible in the image, but stimuli were only delivered through the one electrode in the stratum radiatum, labeled “SC stim”). (B) VSDI data from the stratum radiatum were segmented with the polygonal geometry shown. Black polygonal segments indicate the CA1 segments used for velocity measurements. (C) Raster showing spatiotemporal activity evoked by Schaffer collateral stimulation. The stimulus was delivered at t = 0 ms. Rows, from the bottom to the top, correspond to segments of the hippocampal geometry, from the hilus to CA3 and finally to CA1, as shown in B. For each row, the activation time was computed as the peak in the first derivative of the optical signal. Dots indicate activation times in each row. (D) Velocity measurement. For each CA1 site, the activation velocity in stratum radiatum was computed as the slope of a linear regression of distance versus activation time. The Y axis (distance) was computed as the distance between the centroids of each polygonal segment; centroid positions are indicated with blue dots in A. (E) Average activation velocity. Each point represents one activation velocity measurement. Solid and dashed gray lines show mean activation velocity and standard deviation, respectively. In all recordings, activation velocity was 0.13±0.03 m/s (n = 10). (TIF) [file pone.0108686.s001.tif]

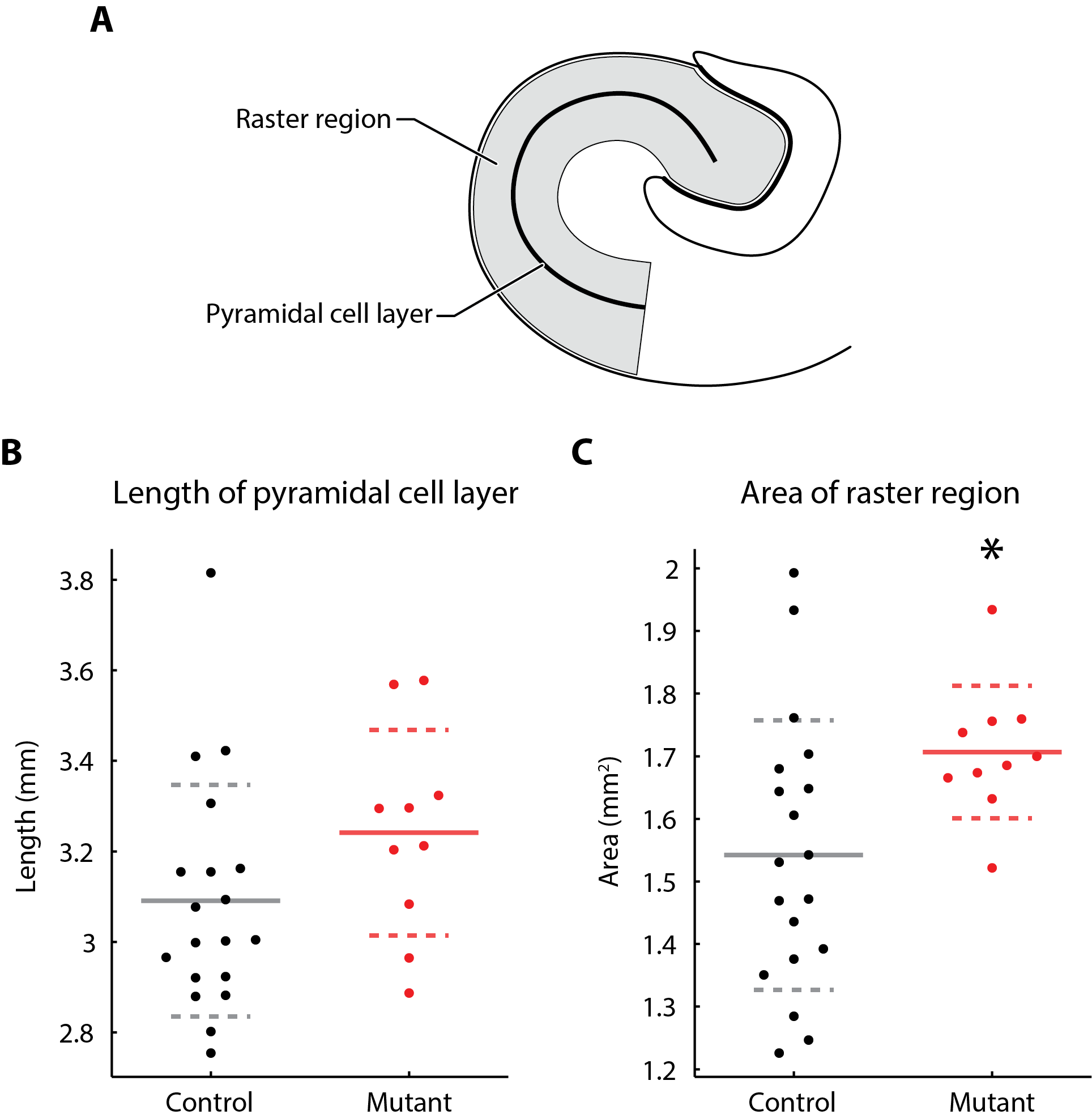

Supplement: Figure S2 — Anatomical comparison using slice geometries. The VSDI toolbox parameterizes the geometry of the slice with a series of polygons to transform the VSDI dataset into a 2D raster plot. Slice anatomy can be quantitatively compared between groups using these polygons. (A) Schematic of analyzed anatomical features. Gray shading indicates the region of the hippocampus parameterized by the toolbox (“Raster region”). The pyramidal cell layer arc is indicated with as a heavy black line. Note that anatomical comparisons were conducted using the original, “un-stretched” polygonal geometries, so that real world units of length (mm) and area (mm2) are preserved. (B) The length of the pyramidal cell layer was not significantly different between control and mutant slices (p = 0.13). (C) The raster area of the hippocampus was significantly different between control and mutant slices (p = 0.03; t-test; n = 19 control and 10 mutant slices). (TIF) [file pone.0108686.s002.tif]

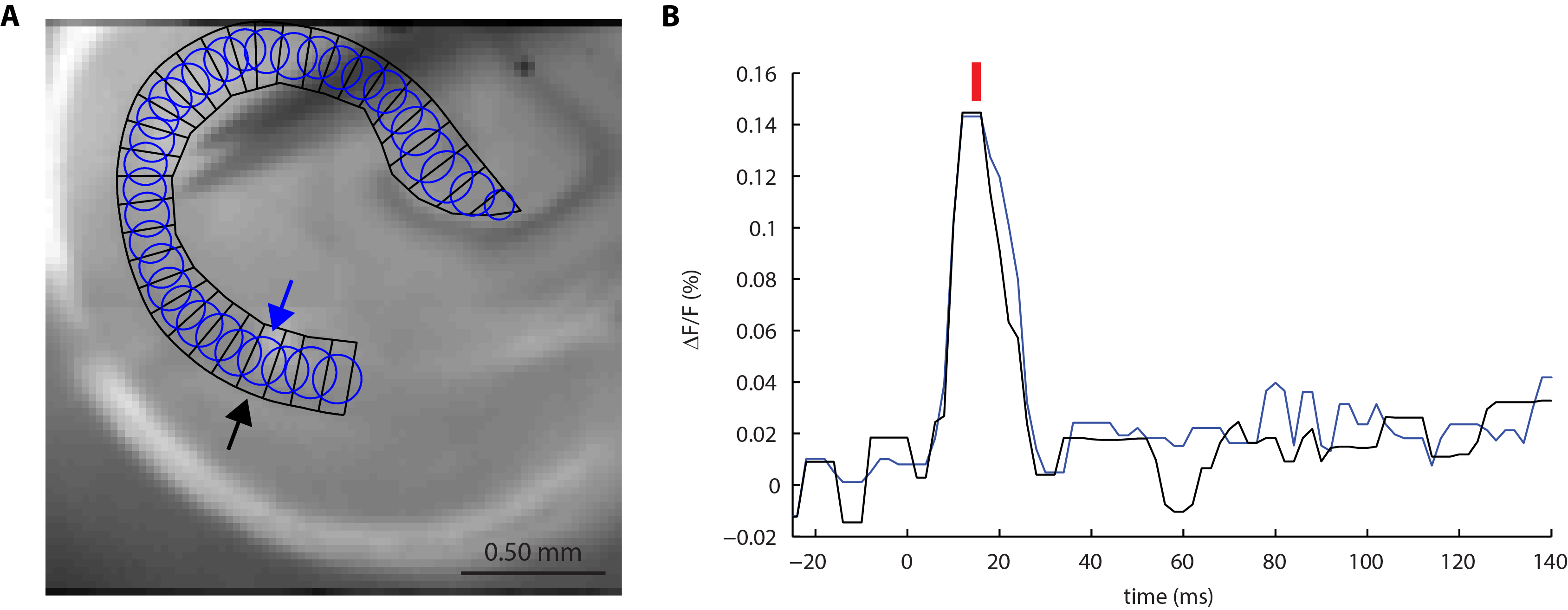

Supplement: Figure S3 — Signal-to-noise analysis. (A) Temporal VSDI signals were obtained from the 3D dataset using anatomically guided image segmentation (black lines), and from conventional, circular image segmentation (blue circles). Each circle has the same area and the same center of mass as its co-localized anatomically guided segment. (B) Temporal signals, obtained by spatially averaging across the area defined by a single anatomical segment (black signal) or circle (blue signal). These signals are from the segment and circle indicated with arrows in panel A. Signal power was measured as the average ΔF/F value during the peak of the excitatory postsynaptic potential (red time interval). Signals were compared to the RMS noise level during the pre-stimulus interval. The signal-to-noise ratio (SNR) was similar when spatial averaging was conducted using either anatomically guided or circular spatial averaging methods (SNRanatomical = 28.5±12.4, SNRcircle = 29.5±13.4; n = 120 regions, paired t-test, P = NS.) This indicates that there is no significant change in temporal resolution when signals are obtained using the new image segmentation method, compared to conventional spatial averaging. (TIF) [file pone.0108686.s003.tif]

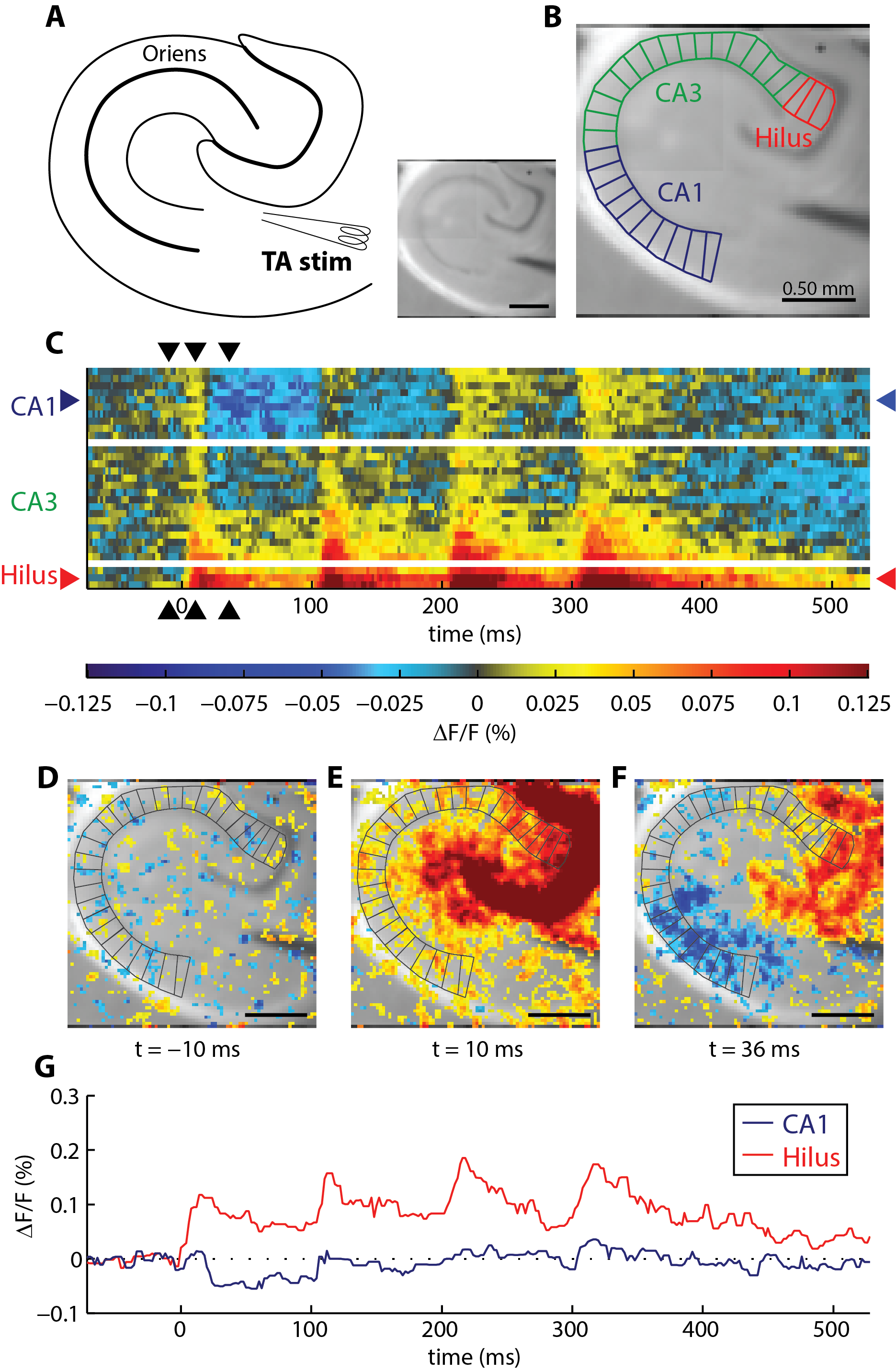

Supplement: Figure S4 — Geometric transformation of activity in stratum oriens, evoked by temporoammonic pathway stimulation. Data are from the same recording as shown in Figure 1 . (A) Schematic and VSDI camera frame showing the hippocampal anatomy and the position of the stimulating electrode. (B) Polygonal geometry for transforming data to 2D. A temporal signal is obtained from each polygon. (C) After transformation, a raster plot completely displays the spatiotemporal response in the stratum oriens. Warmer colors indicate depolarization; cooler colors indicate hyperpolarization. Each row of the raster is a temporal trace from one polygon in B. From bottom to top, rows proceed from the Hilus, to CA3, to CA1. White rows indicate transitions between the anatomical regions. (D–F) Full VSDI camera frames, showing activity at (D) −10 ms (E) +10 ms, and (F) +36 ms (stimulus occurs at t = 0). For comparison, these frames correspond to the black arrowheads that mark the columns in C. To aid visualization of the anatomy in panels D–F, ΔF/F values within 1.5× of the standard deviation of pre-stimulus noise were excluded. (G) Temporal activity at selected hilus and CA1 sites. Spatial positions of these signals are indicated with red (hilus) and blue (CA1) arrowheads in C. (TIF) [file pone.0108686.s004.tif]

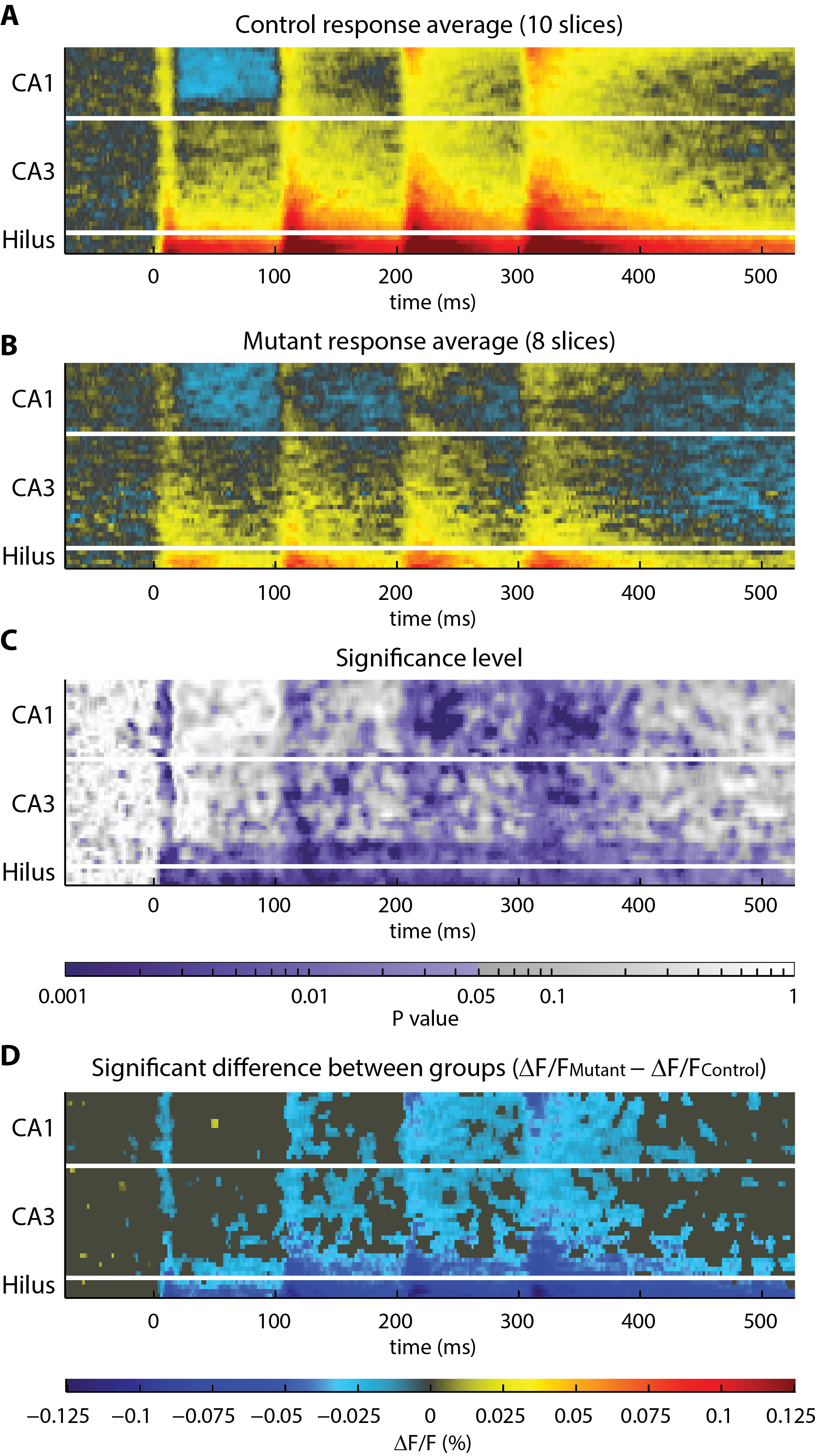

Supplement: Figure S5 — Statistical analysis of the response to temporoammonic stimulation in the stratum oriens. This analysis was conducted in the same manner as shown for stratum radiatum in Figure 2 . (A–B) Visual inspection of the averaged (A) control and (B) mutant rasters suggests that activity is different between groups. (C) Heatmap showing the degree of difference in activity between groups, across space and time. Statistically significant p-values (p<0.05) are shaded purple. The differences seen here in stratum oriens are qualitatively similar to the differences observed in Figure 2 . (D) To obtain a spatiotemporal map of the significant difference in activity in mutant hippocampus, the control raster A was subtracted from the mutant raster B. A threshold was applied to display only sites of significant difference (p<0.05). Significant differences were registered at 6345 of 13244 sites (48%). Color scale is the same in panels A, B, and D. (TIF) [file pone.0108686.s005.tif]

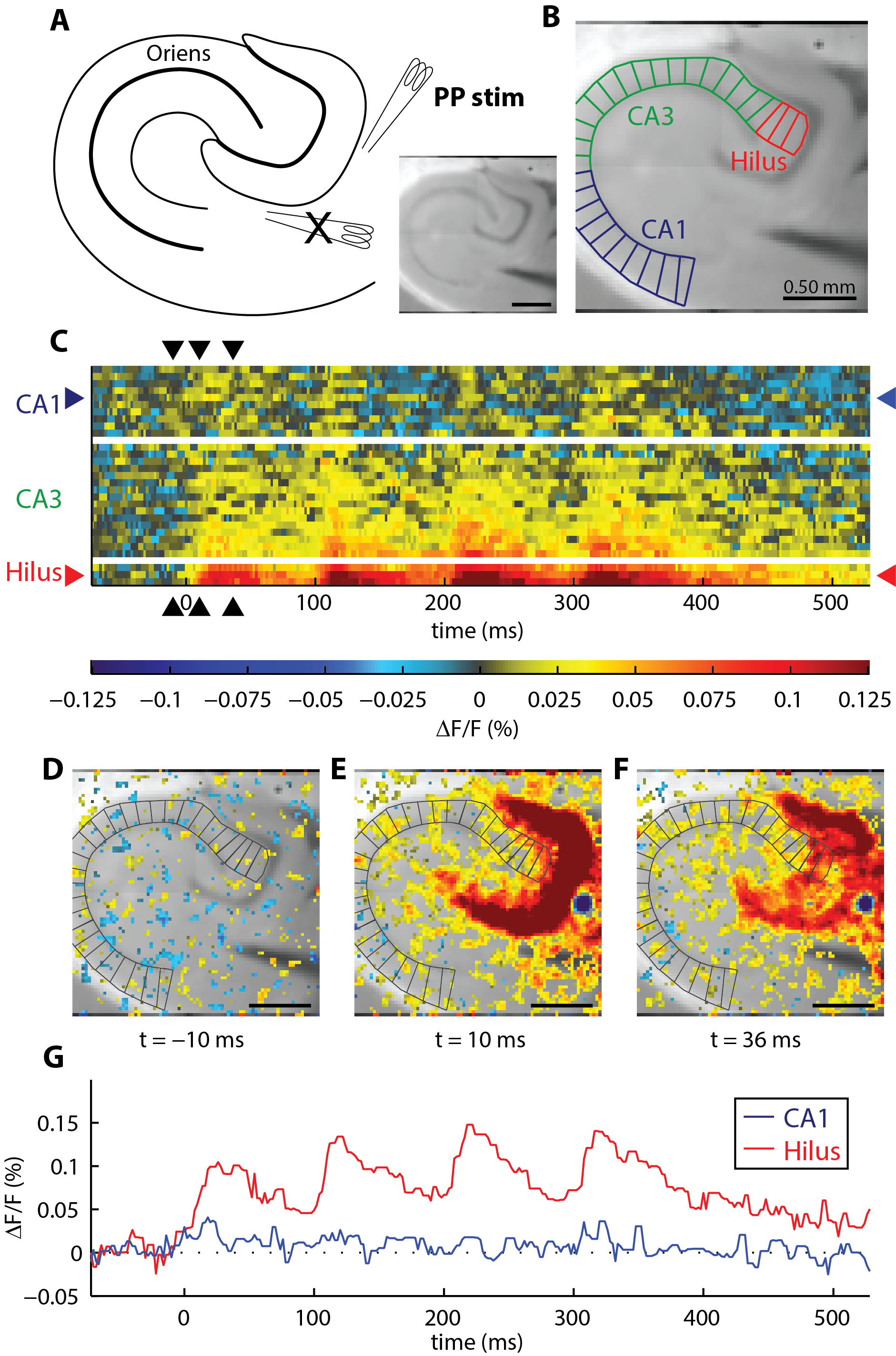

Supplement: Figure S6 — Geometric transformation of activity in stratum oriens, evoked by perforant pathway stimulation. Data are from the same recording as shown in Figure 3 . (A) Schematic and VSDI camera frame showing the hippocampal anatomy and the position of the stimulating electrode. Two electrodes were placed in the slice, but only the electrode labeled “PP stim” was used to deliver the stimulus. The remaining electrode, marked with an “X” in the schematic, was unplugged during this recording. (B) Polygonal geometry for transforming data to 2D. A temporal signal is obtained from each polygon. (C) After transformation, a raster plot completely displays the spatiotemporal response in the stratum oriens. Warmer colors indicate depolarization; cooler colors indicate hyperpolarization. Each row of the raster is a temporal trace from one polygon in B. From bottom to top, rows proceed from the Hilus, to CA3, to CA1. White rows indicate transitions between the anatomical regions. (D–F) Full VSDI camera frames, showing activity at (D) −10 ms (E) +10 ms, and (F) +36 ms (stimulus occurs at t = 0). For comparison, these frames correspond to the black arrowheads that mark the columns in C. To aid visualization of the anatomy in panels D–F, ΔF/F values within 1.5× of the standard deviation of pre-stimulus noise were excluded. (G) Temporal activity at selected hilus and CA1 sites. Spatial positions of these signals are indicated with red (hilus) and blue (CA1) arrowheads in C. (TIF) [file pone.0108686.s006.tif]

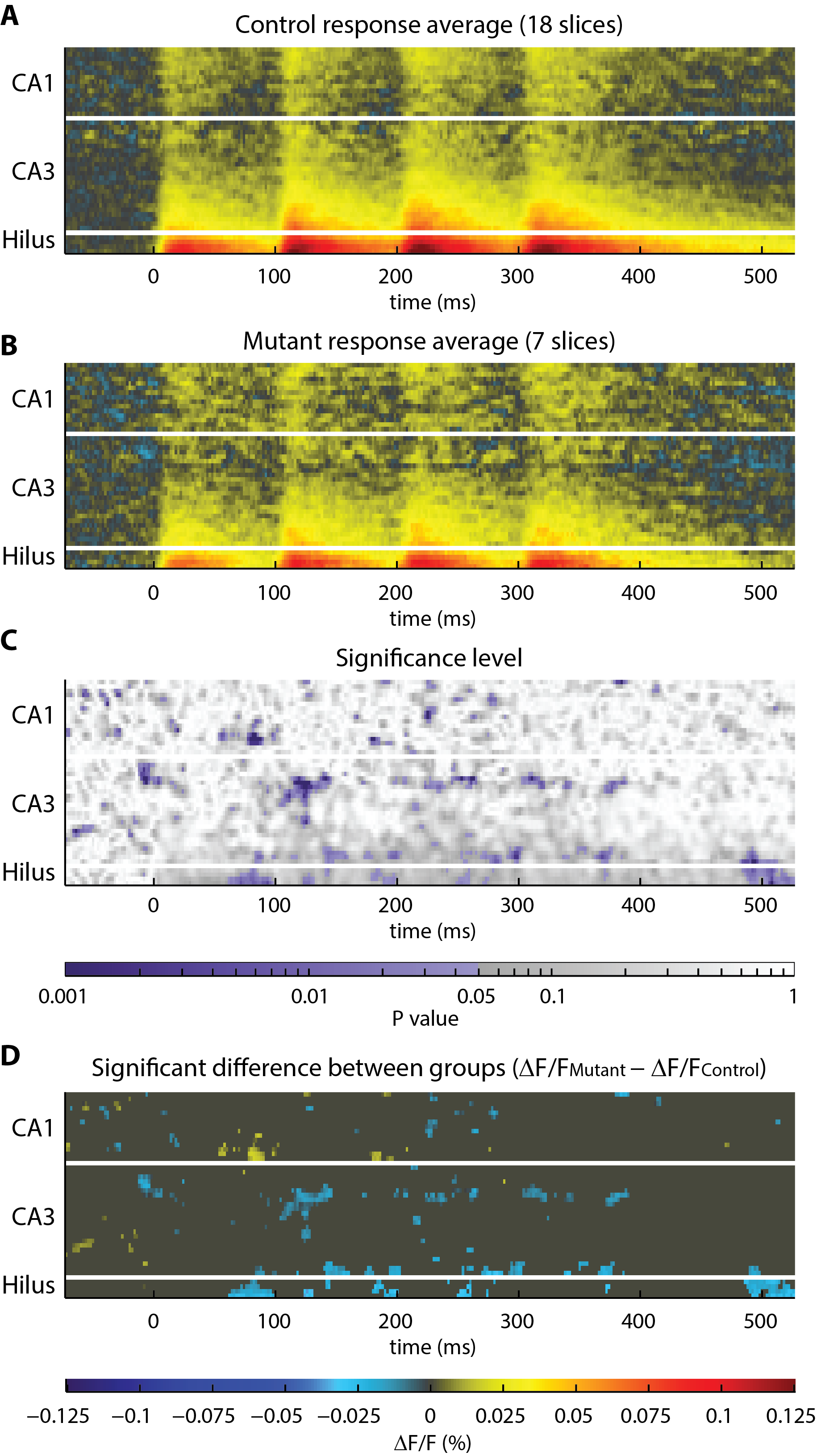

Supplement: Figure S7 — Statistical analysis of the response in stratum oriens to perforant pathway stimulation. This analysis was conducted in the same manner as shown in Figure 2 . (A–B) Visual inspection of the averaged (A) control and (B) mutant rasters suggests that activity is similar in both groups. (C) Heatmap showing the degree of difference in activity between groups, across space and time. Statistically significant p-values (p<0.05) are shaded purple. (D) To obtain a spatiotemporal map of the significant difference in activity in mutant hippocampus, the control raster A was subtracted from the mutant raster B. A threshold was applied to display only sites of significant difference (p<0.05). Significant differences were registered at 717 of 13244 sites (5.4%). Color scale is the same in A, B, and D. (TIF) [file pone.0108686.s007.tif]
