# Supplementary material for: A Toolbox for Spatiotemporal Analysis of Voltage-Sensitive Dye Imaging Data in Brain Slices
Source: PLoS One. 2014 Sep 26;9(9):e108686. doi: 10.1371/journal.pone.0108686 (PMC4178182; doi:10.1371/journal.pone.0108686)
Supplement: Dataset S1 — VSDI toolbox software. (ZIP) [file pone.0108686.s008.zip › VSDItoolbox/supportingInformation2_VSDItoolbox_BourgeoisEB_forSubmit_v093.docx]

**VSDI Toolbox User’s Manual**

**v. 0.93**

**created by EB Bourgeois, updated April 11, 2014**

**Table of Contents**

1. **Installation 1**
2. **Creating raster plots 1**
3. **Grouping rasters into stacks 6**
4. **Co-aligning (“stretching”) rasters to a standard size 8**
5. **Statistical analysis (permutation testing) 9**

**Appendices**

1. **List of included sample data files 11**
2. **Commonly used toolbox commands 12**
3. **Description of values in userSettings.m 15**
4. **Conduction velocity measurement 17**
5. **Anatomical comparison between groups of slices 18**

**Note:** the functionality of the VSDI toolbox is broadly described in our paper:

Bourgeois EB, Johnson BN, McCoy AJ, Trippa L, Cohen AS, Marsh ED. A toolbox for spatiotemporal analysis of voltage-sensitive dye imaging data in brain slices.

For more information on the VSDI Toolbox, please refer to that publication.

**I. Installation**

These instructions have been tested for installation on a Windows machine running Matlab 2010b. The VSDI toolbox will probably work on different systems and with different versions of Matlab, but these haven’t been tested. Also, some commands will have to be modified to work on Mac or Linux. For example, the directory structure on these machines will be delimited with “/” slashes, not “\” slashes.

1. To install the toolbox, extract the zip file to a location on your hard drive, for example, “C:\Users\username\Documents\MATLAB”. Unzipping to this location will create the directory “C:\Users\username\Documents\MATLAB\VSDItoolbox”.
2. Add the VSDI toolbox directory structure to the Matlab path. To do this, run the following command in Matlab’ s command window:

>> path(path,genpath('C:\Users\username\Documents\MATLAB\VSDItoolbox'))

1. Verify that the toolbox works by executing the steps described in **II** through **V** below, using the provided sample data (located in the “VSDItoolbox\SAMPLE_DATA” directory). A list of provided sample data files is provided in *Appendix A1*. After verifying that the toolbox works with the sample data, edit userSettings.m to reflect parameters of your experimental setup. E.g., camera frame rate, pixel size (mm/pixel), stimulus time, etc. The values in userSettings.m are described in *Appendix A3*.

**II. Creating raster plots**

1. Load a voltage sensitive dye imaging (VSDI) data file into the Matlab workspace. The file “VSDIrecording1.mat” (located in “VSDItoolbox\SAMPLE_DATA”) is provided as an example. This file contains two variables:

**D**: a 3-dimensional (3D) matrix of fractional fluorescence values (ΔF/F). (ΔF/F is defined as the change in fluorescent light intensity “ΔF”, relative to the baseline fluorescence level “F”).

**imBg**: a raw camera frame, showing the anatomy of the brain slice. **imBg** is a 2D image. The size of **imBg** must match the size of the first two dimensions of **D**.

1. Convert the data from a 3D movie to a set of 2D raster images using the function **rasterizeMain()**. rasterizeMain() is run from the Matlab command prompt as follows:

>> rasterizeMain(D,imBg);

rasterizeMain() takes 2 inputs: **D** and **imBg** (both described above)**.** rasterizeMain() will prompt the user to outline the boundaries of the regions to convert to 2D, or alternatively, the user can load a pre-existing raster geometry file. Throughout this process, instructions will be displayed in the Matlab command window. You will also be prompted to answer several questions in the command window.

1. **Loading a pre-existing geometry file.** Often, the microscope stage is repositioned between consecutive recordings in a single slice, so that a slice geometry file can be applied to multiple recordings by translating the geometry to align with the slice’s position in the field of view. We have provided utilities to save, load, and translate slice geometries for this purpose.
   - 1. Open a geometry file (.mat) when the “Select a geometry file” dialogue window appears. (Selecting “Cancel” in this window will guide you through the creation of a new geometry file.) A pre-existing raster geometry file is provided for you in “geometrySlice1.mat”. This geometry is for the same slice as the loaded sample data in **D** and **imBg**.
     2. Once loaded, the geometry is shown in Figure 100 (*Figure 1A*).
     3. Translate the geometry to align with the slice by dragging region #1 (the first region defined in the geometry file). For the file provided, this is the CA region (colored blue). Do not drag any of the other regions; they will be translated automatically.
     4. Double click the first region (blue CA region) to translate all of the geometry regions and lines by the same shift that you applied to the first region.
     5. You will be asked if you are happy with the alignment in the Matlab command window. Enter **y** if the regions are well-aligned. To repeat steps *iii* and *iv*, enter **n**.
     6. The polygons for image segmentation are then plotted in Figure 100 (*Figure 1, right*). The data in D will be converted to raster plots, and a “Save rasters as” window will prompt you to save the raster plots to a .mat file. **Note:** the raster data file you save will also contain a backup of all of the geometry variables that were used to segment the data. Accordingly, it is possible to load geometries from geometry files *or* raster data files (in step *i*., above).

**A B**
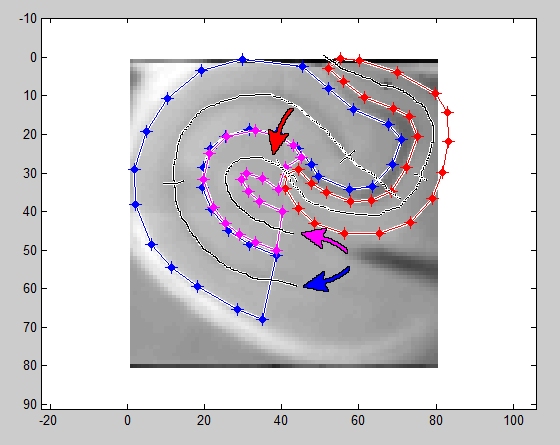

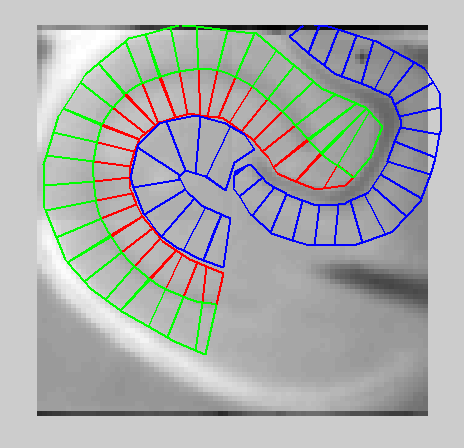
 **Figure 1. A:** geometry lines loaded from “geometrySlice1.mat”. For each region of each slice, the centerline (black) must be drawn in the same orientation: blue, red, and magenta arrows indicate the starting points for the centerlines of the CA, dentate gyrus, and stratum lacunosum moleculare regions, respectively. **B:** polygons used to segment the data into raster plots. The CA region is red and green to indicate that the inside and outside of the centerline arc were segmented into two separate raster plots. The dentate gyrus and stratum lacunosum moleculare are blue, indicating that these regions were combined across the centerline.

1. **Creating a new geometry file.** When the “Select a geometry file” dialogue window appears, select “Cancel”.
   - 1. A message in the Matlab Command Window will instruct you to outline the first region. Note: the order that polygons are drawn in must be the same in each geometry file you create. E.g., you must always outline the CA region first and the dentate gyrus second, so that raster data files will all contain raster arrays in the correct order. To make it easier to remember which region should be drawn first/second/etc., names associated with each numbered region are displayed in the instructions. Region names are defined in userSettings.m.


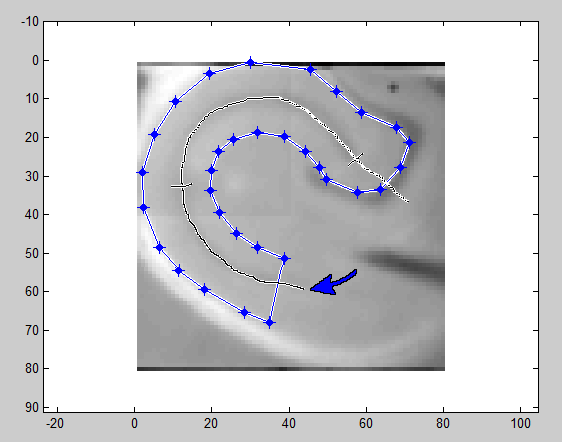

**Figure 2.** Geometry lines drawn for region 1 (hippocampus proper/CA region). The blue polygon was drawn first. The centerline (black) was drawn starting from the subiculum, and proceeding toward the hilus; the blue arrow indicates the starting point of the line. The centerline must intersect with the region polygon at both ends of the centerline.

- - 1. Click to create points of a poly-line to enclose the CA region. After closing the polygon by connecting the line to its starting point, you can drag vertices to adjust the polygon shape. You can also add vertices to the polygon by holding down the ‘a’ key on your keyboard. Double click the region to finalize the polygon.
    2. You will be prompted to draw the region centerline. For the CA region, this line will follow the pyramidal cell layer. Lines must start and finish at the same anatomical positions in each slice. The center line must intersect the region polygon at both ends of the region. For the CA centerline, start from the subiculum (marked with a blue arrow in *Figure 2*) and draw toward the dentate.
    3. You will be asked if you are happy with the line you have drawn. Enter **y** if the line looks good, or enter **n** to redraw the line.
    4. You will be asked how many anatomical divisions you would like to draw. To divide the CA region into 3 sub-regions (the hilus, CA3, and CA1), you will draw two dividing lines: one line at the hilus/CA3 boundary, and one line at CA2. Enter **2**.
    5. Drag to draw a line across the centerline, to mark one of the anatomical boundaries (e.g., the hilus/CA3 boundary). You will be asked if you are happy with the line. Enter **y** if the line looks good, or enter **n** to redraw the line. After you are happy with the first dividing line, you will be prompted to draw more dividing lines until all dividing lines are drawn.
    6. You will be asked if you would like to draw another region. Answer **y** or **n**.
    7. If you choose to draw additional regions, the dentate gyrus region is the 2^nd^ region that will be drawn, and the stratum lacunosum moleculare is the 3^rd^ region that will be drawn. For each of these regions, steps *ii* through *vii* are the same, with the following addenda:
       - 1. When drawing the centerline for the dentate gyrus, start drawing on the enclosed blade (red arrow in *Figure 1A*) and draw toward the exposed blade. When drawing the centerline for stratum lacunosum moleculare, draw from CA1 (magenta arrow in *Figure 1A*) to CA3. The centerlines for these regions should run down the middle of each region and should be roughly parallel to the inner arc of each region. Later, single rasters will be produced from the DG and SLM regions by combining polygons across the region midline (blue polygons in *Figure 1B*).
         2. We use 1 dividing line for the dentate gyrus region, to separate the inner and outer blades of the dentate gyrus. We use 0 dividing lines for the stratum lacunosum moleculare.
    8. After all regions are drawn, you will be asked if you would like to combine each region across the centerline. For the CA region, answer **n**. For the DG and SLM regions, answer **y**. For regions that are not combined across the centerline, the polygonal geometry will be shown in red and green, and you will be asked to verify that the red polygons are on the inside of the arc. If the red polygons are indeed on the inside of the arc, answer **y**. Otherwise, answer **n** (this will switch the names of the inner and outer polygons accordingly).
    9. After all regions are processed, a “Save geometry as” window will appear. Save the geometry file.
    10. The “Save Rasters as” window will appear. Save the rasterized data.

1. After creating the rasters, rasterizeMain() displays the polygonal geometry in Figure 100, and displays the rasters in Figure 101 (see *Figure 3*). The polygonal geometry plot is generated using the rasterPlotPoly() function, as follows:

>> rasterPlotPoly(polyInner,polyOuter,imBg,combineRegions_TrueFalse)

Alternatively, the polygonal geometry can be plotted by providing rasterPlotPoly() a path to a geometry .mat file, as follows:

>> rasterPlotPoly('C:\data\geometrySlice1.mat')

The rasters in Figure 101 are plotted using the function rasterPlotMultiregion() as follows:

>> rasterPlotMultiregion(rasters,rasterNames)

Alternatively, rasters can be plotted by providing rasterPlotMultiregion() a path to a rasters .mat file (a .mat file containing the cell arrays **rasters** and **rasterNames**), as follows:

>> rasterPlotMultiregion('C:\data\geometrySlice1.mat')

**A B

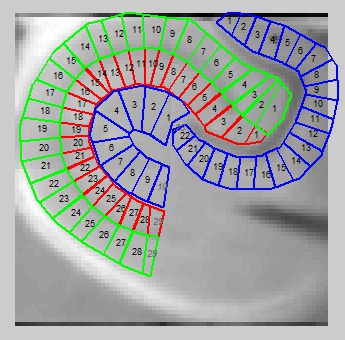

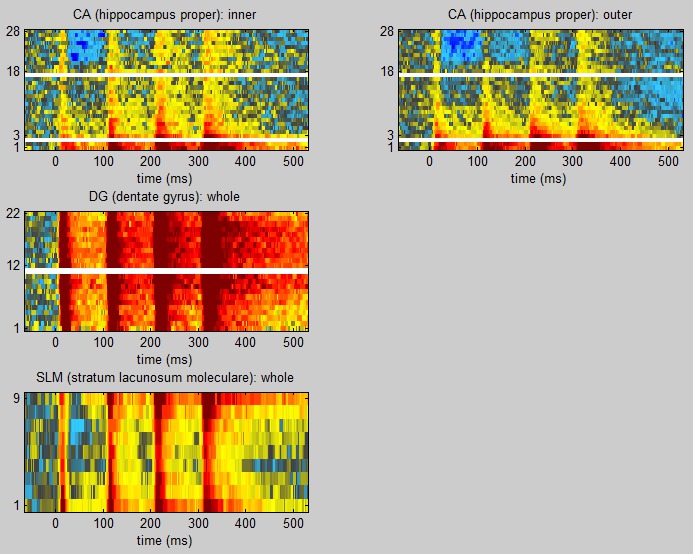

Figure 3. Results from rasterizeMain(). A:** Figure 100 displays the polygonal geometry used to segment the data set. Numbers on each region indicate the row numbers in each raster shown in panel B. **B:** Figure 101 shows all of the raster plots that were generated from the polygonal geometries in panel A. To maintain a correspondence between raster rows and polygon numbers in panel A, the blank (white) rows in panel B are not numbered.

**III. Grouping rasters into raster stacks**

**** Section III is not necessary if you are working with the provided sample data to verify that the toolbox is working. If you are working with the provided sample data, load the file “VSDIta10recordings.mat” and skip to Section IV. ****

- - - 1. After all recordings are processed into raster data files (.mat), we will sort the recordings into groups of interest, e.g., control and experimental groups. We have implemented a simple data management scheme using the functions rasterList_Create() and rasterList_LoadStack(). rasterList_Create() guides the user to generate a list file (.txt) that contains the paths to all of the data files in a group of interest. rasterList_LoadStack() loads the raster data from each data file listed in the list file, and assembles a “raster stack”. A “raster stack” is a cell array of dimensions {n,1}, where n is the number of rasters in the stack, and each cell contains one raster plot.

1. **Creating a list of raster data files with rasterList_Create().** create raster stacks, we first create a list of raster data files using the function rasterList_Create(), called as follows:

>> rasterList_Create;

A load file dialogue is used to select raster data files (.mat) to add to the list. For example, select the provided file “rastersRecording1.mat”. After all of the desired files are added to the list, press ‘Cancel’. The next file dialogue is used to save the list as a text file (.txt). For example, save the list to a new file called “TA10controlGroup.txt”.

Separate lists of rasters should be produced for the control and experimental groups, and the list files should be named to indicate these groupings.

1. **Loading a raster stack with rasterList_LoadStack().** After creating lists with rasterList_Create(), raster stacks will be loaded from these lists using rasterList_LoadStack(), called as follows:

>> DradStackWT = rasterList_LoadStack('C:\Data\TA10controlGroup.txt',1,1);

The first input parameter is a path to the list file. The second parameter specifies which raster to load from each data file. The third parameter specifies whether to load the inner (1) or outer (2) raster from that region. Note that, for combined regions, the “outer” field will be empty. If either the raster number or inner/outer number are not valid for one of the specified raster data files, a warning message will appear indicating that no data were loaded for a given file.

- - - 1. A note on the second and third input parameters for rasterList_LoadStack(): these parameters are indices of the 2D cell array “**rasters**” in each raster data file. For example, “1,1” is used in the above command to tell rasterList_LoadStack() to load the raster **rasters{1,1}** from each data file in the list.

The first number specifies which region to load (e.g., the CA region, the dentate gyrus region, or the stratum lacunosum moleculare region). The second number specified whether to load the inner (1) or outer (2) raster of the region. For regions that are combined across the centerline, only one raster is present. For these regions, the cell for the inner region will contain the raster and the cell for the outer region will be empty.

Each data file contains, in addition to **rasters**, a second cell array called **rasterNames**. **rasterNames** has the same dimensions as **rasters**, and contains the name for the contents of each cell of **rasters**. **rasterNames** is provided to help you to identify which cell(s) you would like to load. For example, in “rastersRecording1.mat”, the name of the data stored in **rasters{1,1}** can be obtained by entering the command:

>> rasterNames{1,1}

This will display the character string “CA (hippocampus proper): inner”. Because the CA region was not combined across the centerline, **rasters** contains two CA raster plots: CA inner and CA outer. CA outer is stored in **rasters{1,2}**.

In the same manner, the name of the data stored in **rasters{2,1}** can be obtained by entering the command

>> rasterNames{2,1}

This will display the character string “DG (dentate gyrus): whole”. Here, the word “whole” is used to indicate that the region was combined across the centerline. For regions combined across the centerline, only 1 raster is generated, and the second cell for the region (here, **rasters{2,2}**) is not used and is left empty. You can verify that this cell is not used by entering the command

>> rasterNames{2,2}

This will display the character string “not used”.

**IV. Co-aligning (“stretching”) rasters to a standard size**

This section assumes you have two raster stacks in the Matlab workspace: one stack, **DradStackWT**, contains control group rasters, and the other stack, **DradStackArxDlx**, contains experimental group rasters. These cell arrays are provided in the sample data file “VSDIta10recordings.mat”, and we will use the names of these arrays throughout this section. Of course, if you are using your own data, you can name the raster stacks as you please.

1. Raster co-alignment is accomplished using the function rasterStackCoalign(). This function takes one input, a raster stack, and returns a raster stack where the rasters in each cell are the same size. rasterStackCoalign() is called to process the control group raster stack as follows:

>> stretchedRadStackWT = rasterStackCoalign(radStackWT);

1. rasterStackCoalign() will display some statistics that describe the number and size of the anatomical regions in each raster in the input raster stack. You will then be prompted to provide interpolated lengths for each region. If a default set of region lengths is found in userSettings.m, those values will also be displayed, and you will be given the option to press enter to accept the set of default region lengths shown. To define your own region lengths, enter a list of integers, enclosed by square braces and separated by spaces, that specifies the desired region lengths.
   For example, **[4 24 16]**.
2. Use rasterStackCoalign() to co-align the rasters for the experimental group in the same manner, using the following command:

>> stretchedRadStackArxDlx = rasterStackCoalign(radStackArxDlx);

It is essential to use the same values for the interpolated region lengths for the experimental group as you used for the control group, so that permutation testing can be conducted to compare the control and experimental groups.

1. Using the following commands, average raster plots can be produced from the stretched raster stacks:

>> radWT_avg= mean(rasterCellStackToMatStack(stretchedRadStackWT),3);
>> radArxDlx_avg= mean(rasterCellStackToMatStack(stretchedRadStackArxDlx),3);

The average rasters permit inspection of the average response in the control and experimental groups. A single raster image (such as the averaged raster **radWT_avg**) can be plotted using the rasterPlotRaster() function, as follows:

>> rasterPlotRaster(radWT_avg,2,70)

In this command, the parameters “2” and “70” were provided so that the raster’s time axis would be rendered in time steps corresponding to the camera’s interFrameInterval (2 ms), and t=0 would be shown on the temporal axis at the stimulus time (70 ms). These parameters are not required to run rasterPlotRaster(), but it is often helpful to adjust the temporal axis in this way to aid in interpreting the data. For more information on plotting rasters, use the command:

help rasterPlotRaster.

**V. Statistical analysis (permutation testing)**

Permutation testing will be conducted in two steps. First, an emperical null distribution will be generated for each site in the spatiotemporal domain, using the function permTestIterate(). Then, statistical comparisons will be conducted at each spatiotemporal site, and the T statisitic for each site will be compared to the empirical null distribution, using the function permTestCompare().

1. The following command will generate an empirical null distribution at each spatiotemporal site:

>> allTs = rasterPermTestIterate(stretchedRadStackWT,stretchedRadStackArxDlx,1000);

rasterPermTestIterate takes three inputs: (1) a co-aligned raster stack for the control group, (2) a co-aligned raster stack for the experimental group, and (3) the number of permutation iterations to perform. A value of “1000” indicates that the null distribution for each site will contain 1000 T values.

1. After null distribution array **allTs** is generated, the following command will test for a significant difference in activity at each spatiotemporal site

>> [tMatReal, diffIm] = rasterPermTestCompare(stretchedRadStackWT,stretchedRadStackArxDlx,allTs,0.05,1)

rasterPermTestCompare() takes 4 inputs: (1) a co-aligned raster stack for the control group, (2) a co-aligned raster stack for the experimental group, (3) the null distribution array allTs (generated by rasterPermTestIterate), (4) the desired alpha level for statistical comparison (here, 0.05), and (5) a binary variable that determines whether the generated **diffIm** should be plotted upon completion of rasterPermTestCompare(). Note: the first two inputs to rasterPermTestCompare(), **stretchedRadStackWT** and **stretchedRadStackArxDlx**, should be identical to the raster stack inputs that were used by rasterPermTestIterate() to generate the null distribution array **allTs**.

1. Two outputs are generated by rasterPermTestCompare():

**tMatReal**: a 2D array containing the T-statistic obtained at each spatiotemporal site, from the comparison of **stretchedRadStackWT** and **stretchedRadStackArxDlx**.

**diffIm**: a 2D array highlighting the significant differences in spatiotemporal activity between groups. This is the difference between the average experimental group response and the average control response, thresholded to show only spatiotemporal sites where a significant p-value was observed (p less than the chosen alpha value).

1. Generate a heat map of p values using the function tStatHeatMapLog(), using the following command:

>> tStatHeatMapLog(tMatReal,allTs,2,70);

The third and fourth inputs to tStatHeatMapLog() define the frame interval (in ms) and the time at which the first stimulus was delivered (in ms). This is similar syntax to what was used to call rasterPlotRaster(). As with rasterPlotRaster(), these numerical inputs are optional. tStatHeatMapLog() can also return a number of output variables that are used for creating a logarithmic color scale bar. For more information on using tStatHeatMapLog(), use the command:

>> help tStatHeatMapLog


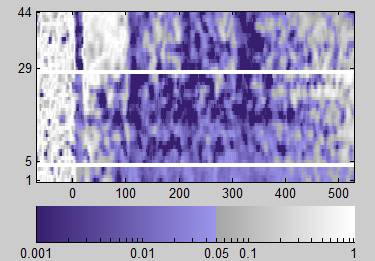

**Figure 4.** Heatmap of P values in the spatiotemporal domain. Purple sites indicate sites of significant difference at an alpha level of 0.05.

**A1: Appendix 1 – List of included sample data files**

*All sample data files are located in the directory “VSDItoolbox\SAMPLE_DATA”.*

geometrySlice1.mat – an example of user-drawn lines that identify the anatomical boundaries of the hippocampus proper, dentate gyrus, and stratum lacunosum moleculare.

geomsAnatomicalAnalysis.mat – example data for comparing anatomical parameters between control and experimental groups of slices.

rastersRecording1.mat – raster plots that were generated by segmenting the slice according to the geometry provided in geometrySlice1.mat. This file also contains all of the geometry variables used to construct the raster plots from the original VSDI movie dataset.

scStim_velocityData.mat – example data for measuring the velocity of impulse conduction with rasterConductionVelocity().

VSDIrecording1.mat – a VSDI recording of a hippocampal slice during temporoammonic pathway stimulation.

VSDIta10_permTestOutput.mat – results from running rasterPermTestIterate() and rasterPermTestCompare() to compare the control and experimental rasters in “VSDIta10recordings.mat” (1000 iterations).

VSDIta10recordings.mat – raster stacks for the control and experimental groups (cell arrays **radStackWT** and **radStackArxDlx**, respectively), and co-aligned (“stretched”) raster stacks for the control and experimental groups (cell arrays **stretchedRadStackWT** and **stretchedRadStackArxDlx**, respectively).

**A2: Appendix 2 – Commonly used toolbox commands**

Creating rasters

>> rasterizeMain(D,imBg);
Transform a 3D video dataset D to a 2D raster plot. A raw camera frame, imBg, is also required.


Data management

>> rasterList_Create
Create a text file containing a list of all raster data files (.mat) for an experimental condition of interest.

>> rasterList_LoadStack;
Load a raster stack from a text file list created by rasterList_Create().

>> stretchedRasterStack = rasterStackCoalign(rasterStack);
“Stretch” each raster in a stack of rasters (in the cell array **rasterStack**) so that all rasters are the same size.


Statistical analysis

>> allTs = rasterPermTestIterate(stretchedRadStackCTL,stretchedRadStackEXP,1000);
Generate an empirical null distribution of T statistics for each spatiotemporal site (**allTs**). “1000” indicates 1000 iterations (i.e., generate 1000 T statistics at each site).

>> [tMatReal, diffIm] = rasterPermTestCompare(stretchedRadStackCTL,stretchedRadStackEXP,allTs,0.05,1);
Compare the control and experimental groups to each other, and determine if the difference is significant by comparing the computed T statistic to the empirical null distribution (**allTs**). “0.05” is the alpha level for statistical comparison.


Plotting tools

>> rasterPlotRaster(rasterImg,2,70)
Plot a raster, using the standard raster color map. “2” and “70” are the camera frame interval and the time of the stimulus, respectively (both in ms).

>> raster_avg = mean(rasterCellStackToMatStack(stretchedRadStackCTL),3);
Average rasters of the same size together to generate a single averaged raster.

>> rasterPlotPoly(polyInner,polyOuter,imBg,combineRegions_TrueFalse)

Plot the polygonal geometry defined in PolyInner and PolyOuter. Alternatively, a polygonal geometry can be plotted by providing rasterPlotPoly() a path to a geometry .mat file, as follows:
>> rasterPlotPoly('C:\data\geometrySlice1.mat')

>> rasterPlotMultiregion(rasters,rasterNames)
Create a multi-panel plot showing all of the rasters in the raster cell array **rasters**. Panels in this plot are labeled with the names in the cell array **rasterNames**. Alternatively, rasters can be plotted by providing rasterPlotMultiregion() a path to a rasters .mat file (a .mat file containing the cell arrays **rasters** and **rasterNames**), as follows: >> rasterPlotMultiregion('C:\data\geomFile.mat').

>> tStatHeatMapLog(tMatReal,allTs,2,70);
Plot a heatmap of P values for all sites in the spatiotemporal domain. **tMatReal** is an array of T values for all sites for the comparison of the control and experimental groups. **allTs** is the empirical null distribution (output from rasterPermTestIterate()). As with rasterPlotRaster(), “2” and “70” are the camera frame interval and the time of the stimulus, respectively (both in ms).

Miscellaneous tools

>> DFoverF = signalDFoverF(D,BG_FRAMES);

signalDFoverF() converts the input **D** to units of deltaF/F. For movies, conversion to deltaF/F is accomplished by dividing all values in each of the temporal signals by the baseline value for that pixel. The baseline value for each pixel is the average value for that pixel during the movie frame numbers specified in **BG_FRAMES**. **D** can be a 4D stack of movies, a single movie, a row of pixels (with dimensions [channels time]), or a single temporal signal (1-D vector). **NOTE:** DFOVERF is returned as type int32 with values equal to **10e7*(deltaF/F)**. This typecasting and scaling is done to avoid “not enough memory” errors.

>> rasterConductionVelocity(rasterCA1rad_scStim,polyCA1rad_scStim,imBg_scStim,50,1);
Conduction velocity measurement. For details, see *A4: Conduction velocity measurement.*

>> rasterCompareAnatomy(polyInnerWT,polyOuterWT,regionPolyCVLWT,regionLinesCVLWT,imBgWT,polyInnerArxDlx,polyOuterArxDlx,regionPolyCVLArxDlx,regionLinesCVLArxDlx,imBgArxDlx)

Anatomical comparison between groups of slices. For details, see *A5: Anatomical comparison between groups of slices.*

>> [VSD, BNC, darkFrame, header] = daopen('C:\data\myDataFile.da');

daopen() loads into Matlab a data file in the RedShirt imaging format. RedShirt Imaging data files have the .da file extension. For a complete description of the data structures returned by daopen(), enter the command: help daopen.

>> rasterBinOut(rasters{1,1},'.\myBinaryOutputFile.bin',1)

rasterBinOut() generates a binary data file for importing into pClamp (Clampfit version 10.3.1.4, Molecular Devices). This will allow you to analyze temporal raster signals with pClamp’s built-in tools for electrophysiological signal analysis (curve fitting, event detection, etc.). To test this function, load the sample data in “rastersRecording1.mat” and execute the command above. The inputs to rasterBinOut are, in order:

1. The raster to export. Note that we specified the raster **rasters{1,1}**, which is the raster for the stratum radiatum.
2. The output file that will be created/overwritten.
3. When nonzero, this input will cause rasterBinOut() to display a list of parameters that will be helpful when using Clampfit’s binary importer interface.

To import data into Clampfit, load the binary file you created (File -> Open Data…), and when the Binary Importer window appears, enter the parameters that were displayed in the Matlab command window.

>> VSDthreshMovie(D,imBg);
VSDthreshMovie() creates a multipage .tif output file, where each page is a frame of the movie. The input **D** is a VSDI recording in ΔF/F units. The input **imBg** is a raw background frame. You will be prompted for a location to save the multipage tif file. You can test this function using the **D** and **imBg** data provided in “VSDIta10recordings.mat”. We recommend using the software package ImageJ to convert the multipage tif file to an AVI movie file. To do this in ImageJ, first load the tif file (File -> Import -> Tiff Virtual Stack…), and then save the movie as an AVI (File -> Save As -> AVI…). You will then be prompted to enter compression and frame rate parameters. We typically use JPEG compression and a 10 fps frame rate.

**A3 – Description of values in userSettings.m**

Variables in userSettings.m are defined in 4 sections: parameters used by rasterizeMain() for raster creation, parameters used by rasterStackCoalign() to coalign rasters, parameters for permutation testing, and parameters for movie output. Brief comments are provided in userSettings.m to briefly describe how to use each variable, here we seek to clarify the usage of several variables that are less straightforward.

**Parameters for creating rasters, used by rasterizeMain():**

GLO_VARS.CVLstep_mm: the desired real-world width of each polygonal segment. This distance is measured along the region’s midline arc. (in mm)

GLO_VARS.regionNamesCVL: this cell array allows you to name the regions you will outline in each recording. The order should be the same in all recordings you analyze, so that analysis can be conducted across all of your subjects directly. The “preallocate” line is present to avoid a warning about changing variable sizes, and is optional. However, it is critical that the value “3” in “cell(3,1)” must equal the number of cells defined in **GLO_VARS.regionNamesCVL**. These values are provided by default:

GLO_VARS.regionNamesCVL =cell(3,1); % preallocate

GLO_VARS.regionNamesCVL{1}='CA (hippocampus proper)';

GLO_VARS.regionNamesCVL{2}='DG (dentate gyrus)';

GLO_VARS.regionNamesCVL{3}='SLM (stratum lacunosum moleculare)';

GLO_VARS.colors: this array allows you to define the color of the poly line you will draw for each separate region. Here, it is critical that the array **GLO_VARS.colors** must have as many rows as the largest number of regions that you will ever attempt to create, so that a color is always defined for every region that is drawn.

GLO_VARS.colors = zeros(50,3); % make the colors array big with a default of black. ("50" makes it possible to outline up to 50 regions without trying to access a nonexistent color)

GLO_VARS.colors(1,:) = [0 0 1]; % CA, blue

GLO_VARS.colors(2,:) = [1 0 0]; % DG, red

GLO_VARS.colors(3,:) = [1 0 1]; % SLM, purple

**Parameters for coaligning (“stretching”) rasters, used by rasterStackCoalign():**

defaultStretchedSizes: this array allows you to define default numbers of rows that each region will contain after interpolation of each region. As with **GLO_VARS.colors**, it is critical that the array **defaultStretchedSizes** must have as many entries as the largest number of anatomical regions that your rasters will ever contain, so that a default set of region sizes is always defined for every raster that is coaligned.

Each “i”th cell in **defaultStrechedSizes{i}** contains i integers, where each integer defines the number of rows to output for each region. When rasterStackCoalign() is provided an input raster that contains 3 regions, rasterStackCoalign() looks for default region sizes in **defaultStretchedSizes{3}**. Using the values defined below, the default region sizes will be 4 rows (region #1), 24 rows (region #2), and 16 rows (region #3):

defaultStretchedSizes = cell(50,1); % preallocate a large cell array, so attempts to access indices >3 do not result in an error

defaultStretchedSizes{1} = 12; % the first cell of defaultStrechedSizes defines the length of the region to interpolate to if only 1 region is found

defaultStretchedSizes{2} = [10 10]; % defines the length of the regions to interpolate to if 2 regions are found

defaultStretchedSizes{3} = [4 24 16]; % defines the length of the regions to interpolate to if 3 regions are found

Edit these values to suit your needs. You also have the option to override these default values at the command prompt when running rasterStackCoalign().

**Parameters for permutation testing, used by rasterPermTestIterate() and rasterPermTestCompare():**

For permutation test iteration and comparison, these values define the size of the kernel for spatiotemporal averaging at each site. Both values must be odd numbers, so that the center pixel of each averaged region is the measurement site of interest.

N_X_SAM = 3; % number of spatial samples to average for measurement at each raster pixel. this value must be odd.

N_T_SAM = 3; % number of temporal samples to average for measurement at each raster pixel. this value must be odd.

**Parameters for movie output, used by VSDthreshMovie().**

GLO_VARS.BGframes: Defines a series of frames of video before delivery of the stimulus. These frames are used to measure the noise level, and this information will be used to define the envelope for noise thresholding.

SD_NOISE_MOV: Noise thresholding is accomplished by rejecting all DF/F values within this number of multiples of the standard deviation of the noise. Higher values of SD_NOISE_MOV will result in more aggressive noise filtering; setting SD_NOISE_MOV to zero will turn off noise filtering.

GLO_VARS.movieSmoothingKernel: Spatial averaging is implemented prior to movie output, to improve the signal/noise in the output movie. A Gaussian smoothing kernel is provided by default. This method of averaging produces, for each pixel, a center-weighted average of the current pixel’s value and its neighbors’ values. Also provided, but commented out, is a command to define a (more conventional) square (5x5) spatial averaging kernel.

% GLO_VARS.movieSmoothingKernel=ones(5,5,1)/(5*5*1); % [r,c,t] smoothing of the movie using convolution (this kernel averages over a 5x5 pixel neighborhood for each pixel)

GLO_VARS.movieSmoothingKernel=fspecial('gaussian',5,1.2); % [r,c,t] smoothing of the movie using convolution (this kernel uses a gaussian kernel to produce a center-weighted average of a 5x5 neighborhood for each pixel)

**A4 – Conduction velocity measurement**

The velocity of the spread of electrical activity can be measured using the function rasterConductionVelocity(). To test this function, sample data is provided in the file “scStim_velocityData.mat”. Load this file into the Matlab workspace and run this command:

>> rasterConductionVelocity(rasterCA1rad_scStim,polyCA1rad_scStim,imBg_scStim,50,1);

The inputs to rasterConductionVelocity are, in order: (1) a raster image of activity evoked by a single stimulus, the polygonal geometry used to generate the raster, (3) a background image for the recording (raw camera frame), (4) the half-window size used for activation time detection, and (5) a debug bit. For more information, on using rasterConductionVelocity, type help rasterConductionVelocity.

**­­­**

**A5 – Anatomical comparison between groups of slices**

The gross anatomy of the control and experimental slices can be compared using the function rasterCompareAnatomy(). To test this function, sample data is provided in the file “geomsAnatomicalAnalysis.mat”. Load this file into the Matlab workspace and run this command:

>> rasterCompareAnatomy(polyInnerWT,polyOuterWT,regionPolyCVLWT,regionLinesCVLWT,imBgWT,polyInnerArxDlx,polyOuterArxDlx,regionPolyCVLArxDlx,regionLinesCVLArxDlx,imBgArxDlx)

This command conducts statistical comparisons to test the hypotheses (1) that the pyramidal cell arcs are different lengths between the control and experimental groups, and (2) that the area of the hippocampus proper is different between the control and experimental groups. A plot showing the distributions of these parameters is also generated (*Figure 5*).

The usage of rasterCompareAnatomy() is fully described in the help section for rasterCompareAnatomy, which you can access with the command help rasterCompareAnatomy.


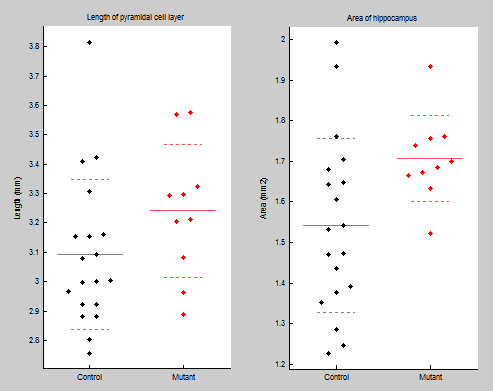

**Figure 5.** Plots of anatomical parameters, generated from the geometric parameters of control and experimental slices by rasterCompareAnatomy().
