# Supplementary material for: A Toolbox for Spatiotemporal Analysis of Voltage-Sensitive Dye Imaging Data in Brain Slices
Source: PLoS One. 2014 Sep 26;9(9):e108686. doi: 10.1371/journal.pone.0108686 (PMC4178182; doi:10.1371/journal.pone.0108686)
Supplement: Supporting Information S1 — Supplemental methods section. (DOCX) [file pone.0108686.s009.docx]

**Supporting Information S1**

***Arx^-/+^;Dlx5/6^CIG^* mice.** All animal protocols were approved by the Institutional Animal Care and Use Committee at the Children’s Hospital of Philadelphia. *Arx^-/+^;Dlx5/6^CIG^* mice were bred, housed, maintained, and genotyped in our laboratory as previously described [[1](#_ENREF_1)]. Briefly, *Arx^-/+^;Dlx5/6^CIG^* mice were generated by crossing female mice possessing two copies of the floxed Arx allele (*Arx^fl/fl^*) with male mice selectively expressing Cre recombinase in *Dlx5/6*-positive neural progenitors (*Dlx5/6^CIG^*). All mice were maintained on a Bl6C57 background. *Arx* is on the X chromosome, and X inactivation theoretically results in roughly half of the interneurons in *Arx^-/+^;Dlx5/6^CIG^* mice expressing the floxed *Arx* allele; the *Arx* gene is selectively deleted in the subset of these interneurons that are *Dlx5/6*-positive. The *Arx^-/+^;Dlx5/6^CIG^* mice exhibit frequent spontaneous seizures and behavioral deficits, recapitulating the phenotype observed in humans with mutations in the *ARX* gene.

**Electrophysiology.** Adult mice were anesthetized with isoflurane and brains were quickly and carefully removed. Hippocampal-entorhinal cortex slices were prepared by bisecting the brain along the midline and then mounting the hemispheres on a vibratome (Leica VT1000S), with the dorsal surface down, at a 12° incline rostral-to-caudal.  An agar block was placed against the midline cut surfaces of the hemispheres to stabilize the brain during cutting. Slices were maintained in artificial cerebrospinal fluid (ACSF) containing, in mM, 130 NaCl, 3 KCl, 1.25 NaH_2_PO_4_, 1 MgCl_2_, 2 CaCl_2_, 26 NaHCO_3_, and 10 Glucose. For cutting, NaCl was replaced with sucrose (sucrose-ACSF).

One parameter in ACSF that can influence the excitability of the preparation is [Mg++]. We use [Mg++] = 1mM because this value is within the physiological range for [Mg++] *in vivo* [[2](#_ENREF_2)]. High [Mg++] can reduce the excitability of the preparation, while low [Mg++] can cause spontaneous bursting [[3](#_ENREF_3)]. Bursting events are not expected to register in our VSDI recordings, for two reasons: (1) VSDI data have poor signal/noise ratio and single-cell bursting events would be difficult to discriminate in pixels that each capture fluorescence from multiple cells, and (2) each recording is in fact an average of 12 separate recordings, so that events out of sync with the stimulus, like bursting events, will be attenuated by between-trial averaging. However, as voltage-sensitive dye technologies improve, it may be possible in the future to discriminate these events from noise.

For recording, slices were maintained at 29°C in an interface chamber (BSC2, Scientific Systems Design, Ontario, Canada). Pathway stimulus strength was set at half the current required to produce a saturating field potential response, as determined by an input-output curve of field potential responses recorded in the stratum radiatum using a glass electrode pulled to a 2-3 MΩ tip, and filled with ACSF. A typical stimulus strength was 200 uA, and stimuli were delivered as 0.1 ms pulses through a bipolar tungsten electrode (model ME12206, World Precision Instruments). For all recordings, four stimuli were delivered at 10 Hz. Field potential recordings were acquired using an Axoclamp 900A amplifier and pClamp 10 software (Molecular Devices).

**VSD imaging.** The VSD di-3-ANEPPDHQ (Invitrogen) was solubilized in 95% ethanol (0.020 mg/uL) and stored at -20°C. Slices were stained with the VSD di-3-ANEPPDHQ (0.067 mg/mL, in ACSF) for 16 minutes. VSD excitation light was provided by 7 high-power green LEDs (Luxeon Rebel LXML-PM01-0100, Philips) coupled to a 535 ± 25 nm filter and 565 nm dichroic mirror. A 610 nm longpass filter further isolated the emitted fluorescence. Using these optics, depolarization of the tissue registered as a decrease in the emitted voltage-sensitive fluorescence. All ΔF/F signals were inverted so that depolarization corresponds to a positive deflection in fluorescence signals, to match with electrophysiological convention. Fluorescence was recorded at 500 frames per second with a fast video camera with 80 x 80 pixel resolution (NeuroCCD, Redshirt Imaging, Decatur, GA). A reverse-lens macroscope with a 50 mm f/1.3 M46 lens was used to image brain slices. With these optics, a 0.025 mm X 0.025 mm tissue region was imaged by each camera pixel (2 x 2 mm full field of view). All recordings were 1.5 s long, with a 10 s delay between recordings to allow fluorescence to recover from photobleaching. Twelve recordings of evoked activity were interleaved with 12 VSDI runs where no stimulus was delivered. Runs without delivered stimuli were used for offline subtraction to correct for any baseline drift over the course of a recording. While more sophisticated de-noising algorithms are available, particularly to handle VSDI noise *in vivo* [[4](#_ENREF_4),[5](#_ENREF_5)], we found that subtraction of unstimulated recordings, combined with 12 trial averaging and 10ms-wide median filtering, produced good noise reduction in our *in vitro* data.

**Statistical analysis across the spatiotemporal domain.** Statistical comparisons were conducted at each pixel in the spatiotemporal raster plots using a permutation test. The logic of the permutation test is that, if the null hypothesis is true (i.e., if there is no difference between groups), then grouping the subjects by genotype will produce no greater difference between groups than grouping the subjects by chance. The permutation test procedure begins by producing an empirical null distribution of t-statistics at each pixel by performing random regroupings of the data. This permutation test was conducted as follows:

1. Each permutation began by pooling the set of control rasters R_control_{1,2,…,m} and the set of mutant rasters R_mutant_{1, 2,…, n} to give one set of all rasters, R_all_{1, 2, …, m+n}. The set R_all_ was randomly shuffled and partitioned into two groups, R_randSetA_ and R_randSetB_. The group R_randSetA_ contained m rasters, and the group R_randSetB_ contained n rasters.
2. The sets of rasters R_randSetA_ and R_randSetB_ were compared to each other at each pixel location pix_i,j_. Note that prior to permutation testing, all of the rasters were converted to a standard size, so that a pixel at position (i,j) in each raster corresponds to the same position in the post-stretching spatiotemporal domain. To reduce noise in the fluorescence values for each pixel, a 3x3 neighborhood of fluorescence values, centered about pix_i,j_, were averaged. A single 3x3 averaged fluorescence value was obtained for each pixel pix_i,j_ in each raster, yielding a total of m and n fluorescence values for each pixel pix_i,j_ in each raster of the two sets of rasters R_randSetA_ and R_randSetB_, respectively. A t-test was conducted to compare the fluorescence values between the sets of rasters R_randSetA_ and R_randSetB_ at each location pix_i,j_. The t-statistic for this comparison was recorded.
3. T-tests were conducted in this way at each location pix_i,j_, to generate an array of t-statistics of the same size as a single raster. This array is the output of a single permutation.
4. The random regrouping and t-statistic calculations performed in steps (1a-1c) were repeated 1000 times, so that 1000 t-statistics were collected at each location pix_i,j_. These 1000 values defined the empirical null distribution of t-statistics for each location pix_i,j_.
5. The original control and mutant raster groups R_control_ and R_mutant_ were compared at each pixel pix_i,j_ in the same manner as described in step (1b-1c). This yielded an array of T-statistics, containing one t-statistic, T_experimentalGroup_i,j_, for each pixel pix_i,j_. T_experimentalGroup_i,j_ was the test statistic to be compared against the null distribution for pix_i,j_.
6. A p-value was computed for each pixel pix_i,j_ by comparing the test statistic T_experimentalGroup_i,j_ from the genotypic group comparison, obtained in step (1e), to the empirical null distribution, obtained in step (1d). A value of T_experimentalGroup_i,j_ in the “tail” of the null distribution indicates a difference between groups that is unlikely to occur by chance. For each pixel, the p-value was computed as the ratio of the number of T statistics in the null distribution greater than the test statistic T_experimentalGroup_i,j_, to the total number of values in the null distribution (in our case, 1000 values). We tested for a difference between groups at a significance level of α=0.05.

**Assessment of permutation test sensitivity.** To compare the results obtained using the permutation test to the conventional statistical approach to VSDI statistical analysis, we compared p-values obtained through permutation testing to p-values obtained using a t-test in equivalent ROIs (**Figure 5**). The spatial regions for conventional VSDI analysis are outlined with magenta and cyan lines in **Figure 5A**; the temporal windows for conventional VSDI analysis are marked with magenta and cyan lines in **Figure 5B-C**. Temporal windows are all 6 ms long (3 camera frames at 500 frames per second). These ROIs correspond to positions in space and time where our *a priori* hypothesis predicted that a functional difference would occur between control and experimental groups.

For each ROI, conventional statistical analysis was conducted as follows: first, the fluorescence values were averaged over the spatial and temporal bounds of the ROI to obtain a single value for each slice. A t-test was used to compare these average values between control and mutant groups. The t-test for each ROI gave a p-value for each ROI. Measurements from each of the 3 ROIs analyzed in this way are shown in the three panels **Figure 5D-F.**

Separately, a p-value was obtained for each ROI by permutation testing. Permutation test p-values were computed for every pixel of the spatiotemporal domain as described in *Statistical analysis across the spatiotemporal domain*, above. ROIs were drawn on the resulting heatmap of p-values (**Figure 5I**) to mark the same spatiotemporal ROIs that were selected for the conventional statistical analysis. The p-values enclosed by each ROI were averaged to yield a single p-value for each ROI. The ROIs for permutation testing, which have equivalent spatial and temporal boundaries with the ROIs used in conventional analysis, are indicated with magenta and cyan rectangles in **Figure 5G-I**. P-values for these tests are reported in the main text.

**Analysis of false positive rate.** We used permutations of control data to assess the frequency at which the permutation test identifies a pixel as significantly different by chance. In a set of control slices that are not expected to be different from each other, the permutation test should theoretically identify differences by chance at the same frequency as the alpha level; in our case, this was 5% for α=0.05. We determined the empirical rate of identifying sites as different by chance, first by using the perforant path dataset because of its larger sample size (n=17 control slices). We measured the rate of chance observations of significance as follows:

1. For each pixel in the spatiotemporal domain, an empirical null distribution was generated by permuting the control data in the manner described above (see *Statistical analysis across the spatiotemporal domain*, steps 1b-1d): each permutation was conducted by dividing the control slices into two random groups R_randSetA_ and R_randSetB_, and a t-test was conducted to compute a t-statistic for each pixel of the spatiotemporal domain. 1000 permutations were conducted to produce a null distribution of 1000 t-statistics at each pixel.
2. The control slices were again shuffled into two random groups. These groups were treated as though they corresponded to an experimental condition of interest. A t-statistic was computed for each pixel pix_i,j_. This t-statistic was treated as the test statistic T_experimentalGroup_i,j_ (described in 1e).
3. For each pix_i,j_, we compared the test statistic T_experimentalGroup_i,j_ to pix_i,j_’s null distribution to compute a p-value (**Figure 6C**).
4. After p-values were computed for all pixels, the total number of pixels that were identified as sites of significant difference (p<0.05) was recorded.
5. The process of shuffling the control slices into two groups and computing p-values for each site, described in steps (2b-2d), was repeated 1000 times. By recording the number of pixels that were identified as significantly different in each iteration, we obtained a distribution of 1000 values that describe the rate of observing differences between groups by chance.

The number of sites identified as significantly different in this test is shown in **Figure 6D-E**. Consistent with the theoretical 5% rate of chance observations of significant difference, this analysis showed that the mean rate of identifying sites of significant difference purely by chance is 4.98%.

We also analyzed the chance rate of observation of significance in VSDI recordings of temporoammonic pathway stimulation in control slices (n=10 slices). For the temporoammonic pathway dataset, we modified the permutation procedure because with 10 slices, only 126 unique combinations of 2 groups of 5 slices are possible. Therefore, R_randSetA_ and R_randSetB_ were not chosen at random but were instead chosen from a list of all 126 possible groupings of the data. For each pixel, a null distribution of 126 t-statistics was produced.

We then selected two groups of slices and treated them as an experimental grouping of interest. This was analogous to (2b) above, but groupings were selected from the list of all possible combinations of 2 groups of 5 slices. For each pixel pix_i,j_, a test statistic T_experimentalGroup_i,j_ was computed and compared to the null distribution for pix_i,j_ to obtain a p-value. All pixels were compared this way to yield a p-value for each pixel, as shown in **Figure 7C.** The total number of pixels identified as significantly different in this comparison was logged. This process was continued for each of the 126 possible grouping of the slices. For each grouping, the number of pixels identified as significantly different was recorded. The distribution of the number of sites identified as significantly different in all 126 combinations is shown in **Figure 7D-E**. In these data, the average rate of identification of sites as significantly different by chance was 3.97%.

**References**

1. Marsh E, Fulp C, Gomez E, Nasrallah I, Minarcik J, et al. (2009) Targeted loss of Arx results in a developmental epilepsy mouse model and recapitulates the human phenotype in heterozygous females. Brain 132: 1563-1576.

2. Slutsky I, Sadeghpour S, Li B, Liu G Enhancement of Synaptic Plasticity through Chronically Reduced Ca2+ Flux during Uncorrelated Activity. Neuron 44: 835-849.

3. Castro-Alamancos MA, Rigas P, Tawara-Hirata Y (2007) Resonance (approximately 10 Hz) of excitatory networks in motor cortex: effects of voltage-dependent ion channel blockers. J Physiol 578: 173-191.

4. Takerkart S, Katz P, Garcia F, Roux S, Reynaud A, et al. (2014) Vobi One: a data processing software package for functional optical imaging. Front Neurosci 8: 2.

5. Fekete T, Omer DB, Naaman S, Grinvald A (2009) Removal of spatial biological artifacts in functional maps by local similarity minimization. J Neurosci Methods 178: 31-39.
